# Faecal microbiota shift during weaning transition in piglets and evaluation of AO blood types as shaping factor for the bacterial community profile

**DOI:** 10.1371/journal.pone.0217001

**Published:** 2019-05-16

**Authors:** Vincenzo Motta, Diana Luise, Paolo Bosi, Paolo Trevisi

**Affiliations:** Department of Agricultural and Food Sciences (DISTAL), Alma Mater Studiorum-University of Bologna, Bologna, Italy; Wageningen Universiteit, NETHERLANDS

## Abstract

The host-microbiota interplay is recognized as a key factor for the homeostatic maintenance in animals. In pigs, the weaning transition represents a drastic changes event leading to high risk of gut dysbiosis, which in most cases results in economic losses for swine industry. The blood type antigens expressed on mucosal surfaces can act as receptors for bacterial adhesion and the hypothesis of possible associations between blood groups and intestinal microbial profiles has been tested in human with contrasting results. Nevertheless, no studies testing the blood type as possible shaping factor for gut microbiota are available for pigs. The results of our previous study suggested the porcine AO blood types system as a possible factor influencing the microbiota composition. In the present study, the changes in fecal microbiota of 12 piglets were followed from 7 days after birth to 2 weeks post-weaning, testing the hypothesis that blood types may impact on its structure. No effects attributable to the difference in blood groups were detected, however, the sampling site (faeces) and the low statistical power might have masked the hypothesized impact. The data clearly showed the rearrangement of the bacterial ecosystem triggered by weaning transition; mainly consisting of a shift from a Bacteroidaceae-Enterobacteriaceae dominated community, to a Prevotellaceae-Ruminococcaceae dominated community. The functional analysis by metagenomic predictions suggested a role of the high levels of long-chain fatty acid in swine milk as energy source for Enterobacteriaceae (*E*. *coli*), in suckling piglets. This study provides a first insight for further investigations; indicating the need for larger sample size, preferably derived from intestinal mucosa, to test the potential effect of blood groups on gut microbiota profiles, and for analyses aimed at assessing the long-chain fatty acids degradation activity within the intestinal microbiota of suckling piglets, with particular attention to the role of *E*. *coli*.

## Introduction

Microbiota stability and colonization of the gastrointestinal tract (GIT) has a crucial role in preserving the host homeostasis and health [1]. Nevertheless, GIT microbiota is variable and exposed to changes based on host genotype, age, exposure to microbes, diet, and many other factors [2–4]. In addition, the shaping of the early GIT microbiota by all these factors may impact the host’s growth performance, immune response and the susceptibility to gastrointestinal disorders such as post-weaning diarrhea, which are key points for animal health and for productive outcomes in swine production [5,6].

The diet seems to be the most important factor affecting the gut microbiota in the short period, but the recent findings on the resilience of the microbiota or part of them, reinforce the idea that other factors can drive the microbiota settlement in the gut. The modification of the gut microbiota is a dynamic event also driven by the continuous cross-talk between the host and the microbiota and it can be modulated by the presence of specific glycoprotein motifs in intestinal mucosa [7].

Since the detection of human ABO blood groups, there has been a constant interest in its potential role in host susceptibility to infectious diseases in human. Indeed, differences on ABO blood groups can affect also in other tissues the antigen expression, which particularly could operate as receptor or co-receptor for bacteria in the intestinal mucus layer [8]. The association between ABO blood group variability and intestinal microbial profile has been analyzed in previous studies on human with contrasting results [9–11]. Mäkivuokko and colleagues [9] reported a higher %G+C in samples from A blood group and the denaturing gradient gel electrophoresis (DGGE) analysis showed different fingerprints in B and AB subjects respect A and O subjects but the taxa responsible for these differences were not identified. Later, Gampa and collaborators [11] conducted a 16S rRNA gene NGS study and attributed the lower presence of %G+C found in the previous study to a lower presence of Lachnospiraceae in subjects with blood group A, they explained the higher presence of Lachnospiraceae in B and O individuals with the activity of β-galactosidases of these bacteria, able to cut the D-galactose residues from the terminal part of the B and O antigen chains. In contrast, the study by Davenport and collaborators [10], conducted on a large sample of 1,500 twins, concluded that there were no differences in the faecal microbial communities associated with the different ABO blood groups.

In pigs, the orthologous of the human ABO blood groups system consists of only one antigen (A) and two blood types (O, A); the OO individuals express the precursor H antigen while the immunodominant structures of A allele (GalNAc α 1–3 (Fuc α 1–2) galactose) antigens characterize the A- pigs [12]. In our previous experience, we observed that the porcine AO groups affected the jejunal mucosa glycomic pattern in pigs challenged with the Enterotoxigenic *Escherichia coli* (ETEC) F4 but also in the non-challenged pigs, suggesting a possible a role of AO system in shaping the microbiota profile in healthy conditions, as already hypothesized for ABO system in humans [13]. Furthermore, in a separate still unpublished trial, we also tested the role of AO genotypes on intestinal glycomic pattern, in a set of ten-week-old healthy pigs. The immunohistochemistry analysis highlighted a significant effect of AO genotypes on fucosilation profile of the jejunal mucosa (brush border, villi and crypts goblet cells), revealing a higher immunoreactivity to Ulex europaeus agglutinin I (UEA; fucose-specific) for the OO genotype compared to AO genotype in all tested sites (personal communication, Luise et al. 2018).

However, to the best of our knowledge, very little is still known about AO influences on bacteria community in pigs.

The aims of the present study are: i) test the hypothesis that the genotypes for AO blood groups impact on piglets’ fecal bacterial community long some crucial moments such as suckling and weaning; ii) enrich the knowledge about the development of the early-life microbiota in piglets, contributing to identify potential key points in its shaping.

## Material and methods

The procedures were conducted in compliance with Italian laws on experimental animals and approved by the Ethic-Scientific Committee for Experiments on Animals of the University of Bologna, ID number 704.

### Animals and sampling

The samples were collected from a commercial farm located in the northern Italy, in the area of PDO Parma ham. The sampled pigs were a commercially available cross breed pigs (*Sus scrofa*) Landrace x Large White x Duroc. The sows were fed a commercial corn-based diet that meets the nutritional requirements recommended by the NRC 2012. Sows and suckling piglets were reared in conventional farrowing cages of 4.5 m^2^, and the farm respected the Council Directive 2008/120/EC of 18 December 2008. The sows were fed with a liquid diet following the feeding curve recommended from the genetic company with a max amount of 6.5 kg of feed /day. The water was freely available for sows and piglets during all the suckling period by a nipple.

Bristles were sampled from animals for genomic DNA extraction. Several sows were screened for the AO genotype. Two sows with AO and two with OO genotypes were selected. For each sow, three female piglets with blood group genotype identical to the mother were chosen and followed until 2 weeks post-weaning. By choosing more than one sow per genotype group we intended to limit the possible confounding effect of litter origin. To limit the impact of other confounding factors, the sows and their litters were reared in the same batch during the lactation period, the same creep feed was provided after the second fecal sampling (day 14), and at the weaning (day 28) the piglets were moved to the same box. The enrichment material consisted in pieces of fresh wood suspended in horizontal position below snout level, replaced at regular intervals to ensure a sufficient level of smell and freshness. The room temperature was controlled and the access to water was guaranteed ad libitum.

From each piglet, fecal-swabs were collected at day 7 (timepoint I, tI), day 14 (timepoint II, tII) after birth and 2 weeks after weaning (timepoint III, tIII). Individual samples were also collected by fecal-swab from the sows in the pre-weaning period (tI and tII)—in which the piglets were still breastfed and in contact with the mother’s feces—in order to have a “maternal reference” microbiota.

The samples were immediately inserted into sterile tubes, frozen in liquid nitrogen and stored at -80°C until use.

### Blood groups genotyping

The porcine DNA was extracted from bristles by using an established protocol in our laboratory, modified from Zabek et al.[14]. In brief, the bristle bulbs were incubated in Proteinase K solution (10 mg/mL of proteinase K in buffer [20 mM Tris HCl (pH 8.4), 50 mMKCl]) for two hours at 50°C, then the proteinase was inactivated at 95°C for 10 min, the samples were briefly spun in a microcentrifuge, the supernatant solution containing the animal DNA was transferred to a new tube and stored at -20°C until use. The multiplex PCR for AO blood groups identification was performed as described in Nguyen et al. [15], using the primers reported in S1 Table.

### Bacterial DNA extraction and sequencing

Bacterial DNA was isolated and extracted with FastDNA SPIN Kit for Soil (MP Biomedicals, Santa Ana, Ca, USA) following the manufacturer’s instructions. Quality and purity of the isolated DNA were checked by spectrophotometry on the NanoDrop (Fisher Scientific, 13 Schwerte, Germany).

The V3-V4 hypervariable region of the 16S rRNA gene amplicons were produced using thee primers Pro341F: 5′-TCGTCGGCAGCGTCAGATGTGTATAAGAGACAGCCTACGGGNBGCASCAG-3′ and Pro805R: 5′GTCTCGTGGGCTCGGAGATGTGTATAAGAGACAGGACTACNVGGGTAT

CTAATCC-3′. The libraries were prepared using the standard protocol for MiSeq Reagent Kit v3 and sequenced on MiSeq platform (Illumina Inc., San Diego, Ca, USA).

The raw reads obtained are publicly available at the European Nucleotide Archive (ENA) under the accession number PRJEB23858.

### Bioinformatics and biostatistics

Two piglet samples (one for genotype AO and one for genotype OO from timepoint I) were excluded from analysis for insufficient yield in sequencing process (less than 1000 reads). The reads from remaining 42 samples were analyzed using subsampled open reference OTU strategy in QIIME v1.9.1 [16] following authors’ recommendations. In brief, the paired-end reads were merged, demultiplexed and quality filtered with a cutoff of Q20. The subsampled open-reference OTU-picking strategy was performed using uclust with 97% sequence similarity. The chimeric sequences were identified and removed using the “Blast_fragments approach”. The representative sequences were assigned taxonomy against the Greengenes database V13_8 using uclust with a 90% confidence threshold and the singleton and low count OTUs were removed with a threshold of 0.005% [17].

Finally, in order to infer the functional profile of the bacterial community, the OTU table was used to perform metagenome prediction in PICRUSt 1.1.0 [18] following authors’ recommendations. Briefly, starting from the open reference OTU table constructed in QIIME a new closed reference OTU table was generated using the gg_13_5_97 Greengenes database as reference, the OTU table was normalized for 16S rRNA gene copy number and the metagenome functional prediction was obtained applying “predict_metagenomes” procedure, then, the “metagenome_contribution” procedure was applied to determine the OTUs contributing to particular functions.

The OTU table was imported in R 3.3.2 for the ecological parameters evaluation. The variability within bacterial communities (alpha diversity) was assessed with the Shannon index in Phyloseq package [19] and the effect of genotype and litter were tested with a mixed model in nlme package fitting the models reported in the S1 Models. The global differences among bacterial communities (beta diversity) were assessed by Bray-Curtis distance matrices in Vegan package [20], and plotted with a Non Metric Multidimensional Scaling (NMDS) approach. The effects of genotype, litter and time were tested with *adonis* procedure implemented in the same package, fitting the models reported in S2 Models.

In order to test taxonomic differential abundances, the family aggregated data were normalized by cumulative sum scaling approach and analysed in metagenomeSeq package [21], the effect of genotype was tested with the procedure for longitudinal data “fitTimeseries”[22] and the “fit-zig model” implemented in the same package was used for the other pairwise contrast.The level of significance was defined by p values (P) <0.05. For multiple comparisons the Benjamini& Hochberg correction was applied (Padjust).

For the metagenomic predictions, we focused our attention on the functional changes in microbial community of piglets, we analyzed the pathway aggregated data (level3) in order to have a general vision on the metabolic shift in bacterial community, then, the entire dataset of KEGG Orthology genes (KOs) was tested to have a deep resolution within the pathways.

The difference for Pathway aggregated data were tested in STAMP software with Welch’s test, the level of significance was defined by Padjust<0.05. The whole predicted KOs table was imported in R 3.3.2 and the differential abundances of KOs between pre and post-weaning period were assessed in DeSeq2 package using the Wald test, the significance was defined by Padjust< 0.01, the resulting differences were plotted in a metabolic map with iPath2 [23] application after excluding the pathway conflicts.

The R package “micropower” [24] was used to estimate (*a posteriori*) the statistical power of the study. In brief, starting from PERMANOVA (*adonis*) results the group-level effects size is quantified by the adjusted coefficient of determination omega-squared (*ω*^2^), then, the method simulates a set of pairwise distances according to a prespecified within-group distance (derived from a reference population) and allows to estimate the statistical power and the effect size, subsampling different samples size with a bootstrap procedure. In our case, the actual effect size of the trial was calculated for the time factor and then for the genotype factor in the three timepoints. Afterwards, 100 distance matrices (Bray-Curtis) were simulated using an average within-distance of 0.4 and, for the estimation of the power and effect size, 100 bootstrap iterations were performed using an alpha value of 0.05.

To refine the taxonomic classification, the raw reads were processed using the R package “DADA2” (version 1.10). The Divisive Amplicon Denoising Algorithm (DADA) unlike the OTUs-based approach, that typically uses a 97% identity level to cluster reads in taxonomic units, is based on the identification of single nucleotide sequence variants which are imperceptible to OTU methods [25]. The DADA2 pipeline was applied with default settings, using SILVA (release 132) database [26] for the taxonomic assignment.

## Results

### Alpha and beta diversity

A total of 3,445,968 reads were attributed to 1,439 total OTUs distributed among samples as shown in S2 Table, the relative rarefaction curves are reported in S1 Fig, showing the tendency to the plateau in all samples which suggests that the sampling effort (sequencing depth) was adequate to describe the variability within the microbial communities analysed.

**Fig 1:**
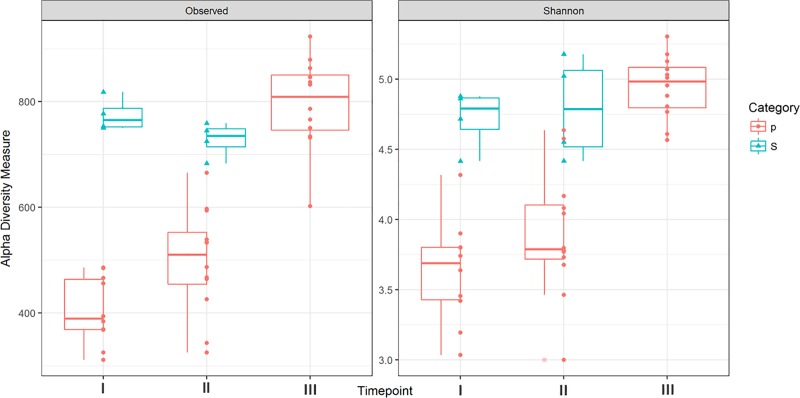
Box plot of observed OTUs abundances and Shannon index values. Category: p = piglets, S = sows. Timepoint: I = day 7 post-farrowing, II = day 14 post-farrowing, III = day 14 post-weaning.

The data showed an increasing alpha diversity in piglet samples along the time (lm, r^2^ = 0.73, P<0.001), with post-weaning values (timepoint III) comparable to the alpha diversity observed in sows’ microbial community (Fig 1).

**Fig 2:**
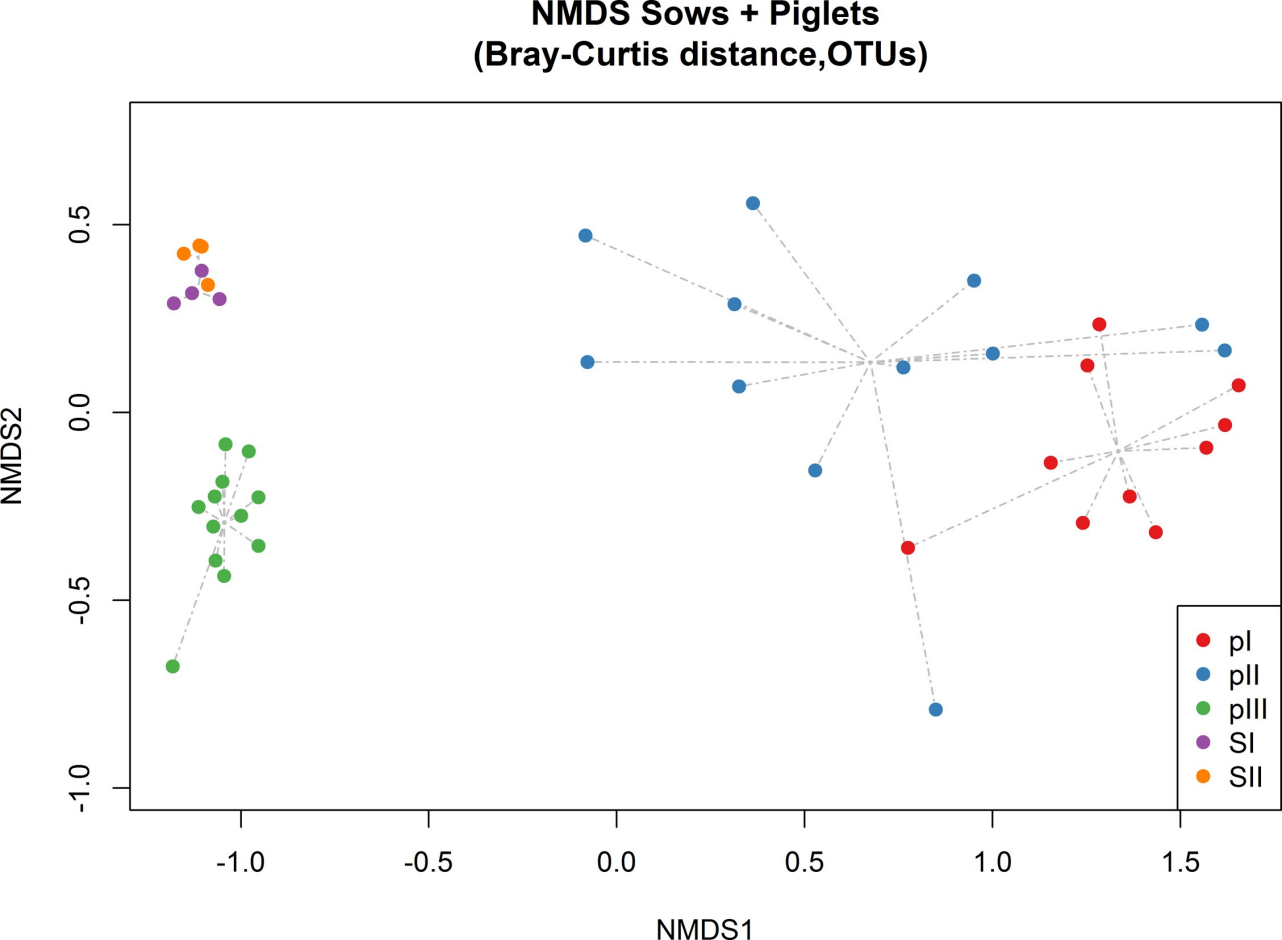
Non-metric multi dimensional scaling (NMDS) on Bray-Curtis distances at OTUs level. pI = piglets timpoint I (day 7 post farrowing), pII = piglets timepoint II (day 14 post farrowing), pIII = piglets timepoint III (day 14 post weaning), SI = sows timepoint I (day 7 post farrowing) SII = sows timepoint II (day 14 post farrowing).

No significant differences were reported for genotype and litter factors on alpha diversity in piglets (S1 Models). Concerning the beta diversity, a variation of the bacterial communities composition was observed over the time according with the age of the animals (Fig 2) (adonis r^2^ = 0.42, P = 0.001), no significant differences were reported for the genotype and litter factors (S2 Models).

### Taxonomic composition

We found that a large part of the reads—in average 53% in sows and 41% in piglet samples—was not classified at the genus level. On the contrary, the family level showed a better coverage -92% of reads in piglets and 71% of reads in sow samples- in taxonomic classification of the reads (S2–S4 Figs) and represents a good compromise to associate the taxonomic composition of a bacterial community with roles taxa-associated in bacterial ecosystem. Furthermore, the beta diversity on family aggregated data revealed a pattern compatible with that observed at OTUs level (S5 Fig).

**Fig 3:**
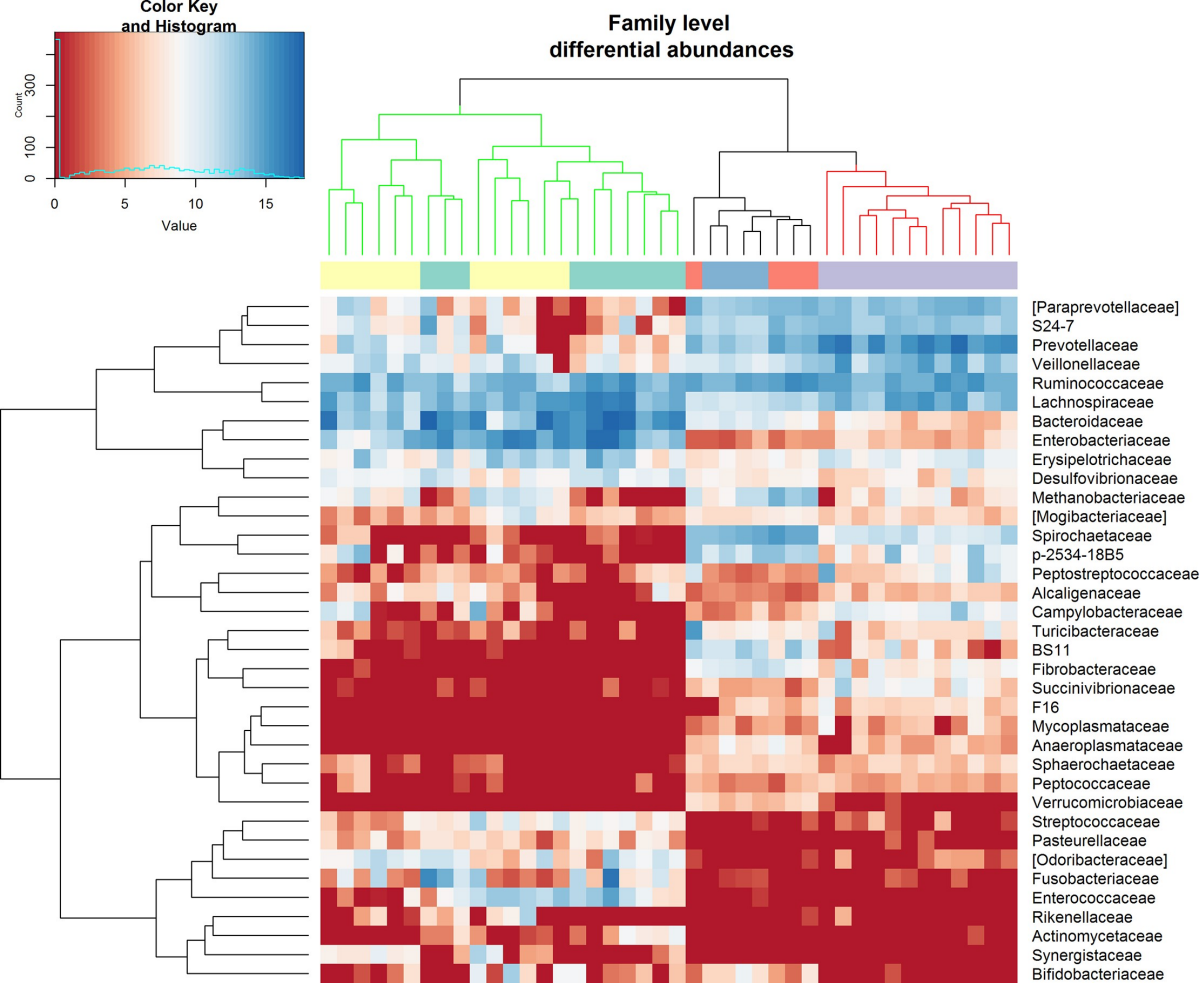
Family Heatmap and hierarchical clustering. The listed families showed significant differential abundances between suckling piglets and weaned piglets and between weaned piglets and sows (see also S7 and S8 Figs). Upper bar: green = piglets timepoint I; yellow = piglets timepoint II; purple = piglets timepoint II, orange = sows timepoint I; blue = sows timepoint II. Upper dendrogram: green branches = pre-weaning piglets; red branches = post-weaning piglets; black branches = sows. The family abundances were normalized by cumulative sum scaling and log2 transformed in metagenomeSeq package.

Regarding differential abundance analyses, a total of 40 families were identified, for 30 of these differential abundances were reported between pre- and post-weaning period (Padjust<0.05), whereas (Fig 3 and S7 Fig),

17 families were found differentially abundant between bacterial community of weaned piglets and sows (Fig 3 and S8 Fig), and only 12 non-dominant families were differentially abundant between timepoint I and timepoint II in piglets (S6 Fig), indicating that weaning causes the most significant change in the bacterial community. The *fit timeseries* test did not show significant differences for the genotype factor. The major shift (in term of relative abundances) due to the weaning concerned the increase in Prevotellaceae (1.5% pre-weaning; 27% post-weaning) and the decrease of Bacteroidaceae (25% pre-weaning; 0.08% post-weaning) and Enterobacteriaceae (15% pre-weaning; 0.05% post-weaning) families (S7 Fig). For the comparison between the faecal microbiota of weaned piglets (timepoint III) and that of the adult pigs (Sows), the principal differences in dominant families concerned a greater presence of Spirochetaceae (11.26% sows; 1.20% weaned piglets) and Ruminococcaceae (16.94% sows; 10.75% weaned piglets) in sows, conversely Prevotellaceae (27% weaned piglets; 5.10% sows) and Lachnospiraceae (8.35% weaned piglets; 1.63% sows) resulted with greater abundances in weaned pigs respect to the sows (S8 Fig).

### Metagenomic prediction

In order to analyse the shift in metabolic potential of the bacterial community we focused our attention on differences related to the weaning transition in piglets. From a total of 232 “level3” KEGG pathways that were present in the samples, 165 revealed significant difference (Welch’s T test, Padjust<0.05) between pre- and post-weaning, on the contrary, only eight pathways (most of which are not related to bacterial metabolisms) showed significant differences between timepoint I and II (S9 Fig), confirming the major shift between pre- and post- weaning. No significant differences were reported between the two genotypes in the different timepoints. Among the pathways data it is possible to note that the microbiota genes encoding for proteins related to the fatty acids and galactose metabolisms would be more represented in the microbial communities during the lactation phase, whereas, in the post-weaning an increase in those related to starch and sucrose metabolism is noted (S10–S12 Figs).

**Fig 4:**
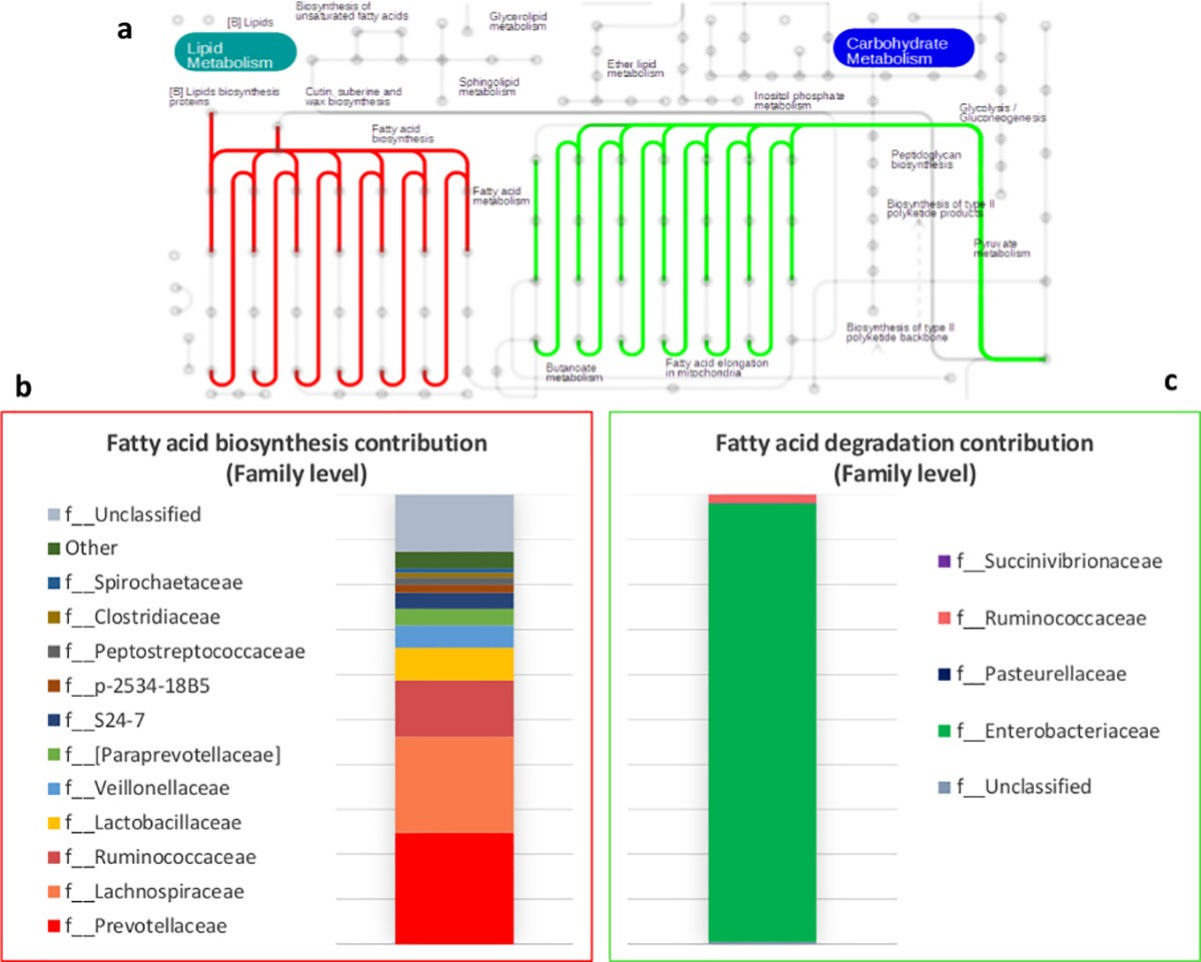
Lipid metabolism in bacterial community of the piglets. Differences between pre-weaning (green lines and square) and post-weaning (red lines and square) and taxonomic contribution at family level. For the complete metabolic map see S13 Fig.

To better dissect the effect of weaning within the pathways we analysed the entire set of KOs predicted genes (4,697 KOs) testing for differences between pre- and post-weaning: 3,018 KOs reported significant differences (Padjust<0.01); 1,152 KOs mapped successfully in iPath2 maps and 406 of these belonged to the central metabolic pathway map (S13 Fig).The representation through the central metabolic pathway map allowed us to visualize and isolate an interesting pattern within the lipid metabolism (Fig 4) among this large dataset.

The difference shown in Fig 4 were determined by KOs associated to fatty acid degradation (*fad*), which were enriched in pre-weaning microbiome, and by KOs associated to fatty acid biosynthesis (*fab*) which were enriched in post-weaning microbiome (Table 1).

**Table 1:** List of predicted genes involved in lipid metabolism differentially enriched in fecal bacterial community of weaned piglets (Post-Weaning) and suckling piglets (Pre-Weaning).

KO id*	Gene	Module	Group	baseMean^a^	log2FC^b^	lfcSE^c^	Stat^d^	Padj^e^
K01782	fadJ	M00087 (Fatty acid degradation, beta-Oxidation)	Pre-Weaning	2390.19	3.39	0.37	9.22	1.28E-19
K01825	fadB	M00087 (Fatty acid degradation, beta-Oxidation)	Pre-Weaning	3165.73	6.08	0.44	13.79	4.21E-42
K00632	fadA, fadI	M00087 (Fatty acid degradation, beta-Oxidation)	Pre-Weaning	1539.78	2.74	0.36	7.54	1.47E-13
K00645	fabD	M00082 (Fatty acid biosynthesis, initiation)	Post-Weaning	16205.79	-0.55	0.10	-5.81	1.58E-08
K09458	fabF	M00083 (Fatty acid biosynthesis, elongation)	Post-Weaning	17326.54	-0.56	0.07	-7.79	2.19E-14
K00059	fabG	M00083 (Fatty acid biosynthesis, elongation)	Post-Weaning	34189.71	-0.41	0.08	-5.35	2.09E-07

*Enzyme definition: **K01782** = 3-hydroxyacyl-CoA dehydrogenase / enoyl-CoA hydratase / 3-hydroxybutyryl-CoA epimerase; **K01825** = 3-hydroxyacyl-CoA dehydrogenase / enoyl-CoA hydratase / 3-hydroxybutyryl-CoA epimerase / enoyl-CoA isomerase; **K00632** = acetyl-CoA acyltransferase; [acyl-carrier-protein] S-malonyltransferase; **K00645** = [acyl-carrier-protein] S-malonyltransferase; **K09458** = 3-oxoacyl-[acyl-carrier-protein] synthase II; **K00059** = 3-oxoacyl-[acyl-carrier protein] reductase

**a** = mean of genes counts normalized for sequencing depth

**b** = log2 Fold Change

**c** = log2 Fold change standard error

**d** = Wald statistic value

**e** = Benjamini-Hochberg adjusted p value

The identification of the OTUs contributing to these functions showed that Enterobacteriaceae is the family mainly involved in fatty acid degradation whereas Prevotellaceae, Lachnospiraceae and Ruminococcaceae are the main contributors to the fatty acid biosynthesis (Fig 4).

The taxonomic refinement by DADA2 pipeline revealed that the Enterobacteriaceae were almost entirely represented by the genus *Escherichia*/*Shigella* (S14 Fig).

### Blood type effect and statistical power

As already mentioned, the genotypes determining the AO blood groups did not seem to influence the structure of faecal microbial communities neither in terms of alpha (mixed model P = 0.58) nor in terms of beta diversity (PERMANOVA P = 0.33). In addition to an actual absence of the genotype effect, the absence of statistically significant differences could be due to the limited statistical power of sampling carried out in the present study.

**Fig 5:**
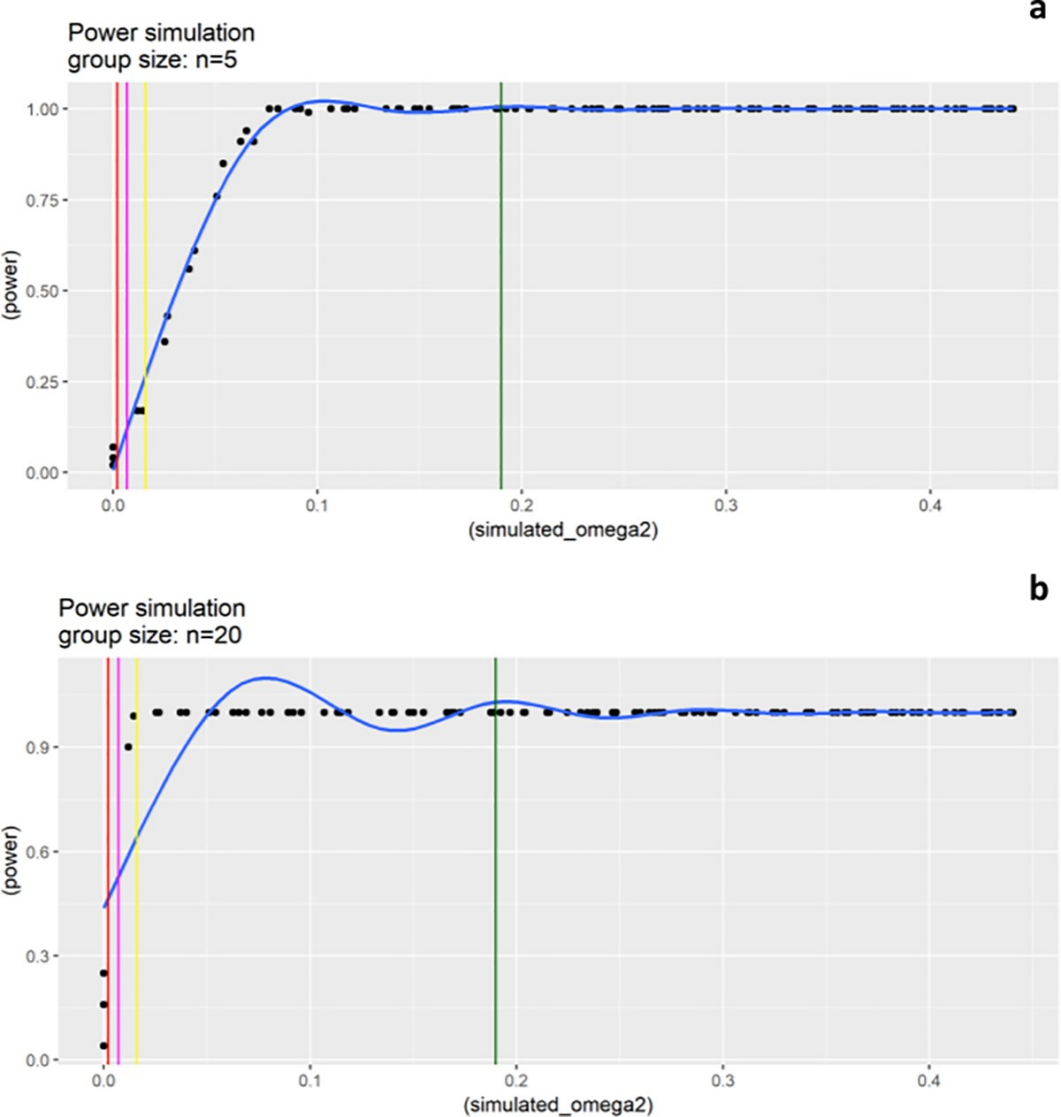
**Power/Effect size (omega2) simulations with a sample size of n = 5 (a) and n = 20 (b) per group.** The colored vertical lines represent the *ω*^2^ values calculated on our experimental groups; dark green = Effect size for timepoint factor, yellow = Effect size for genotype factor at timepoint I, magenta = Effect size for genotype factor at timepoint III, red = Effect size for genotype factor at timepoint II.

The calculation of the effect size returned an *ω*^2^ value of 0.195 for the timepoint effect, whereas, the *ω*^2^ values for the genotype effect were 0.016, 0.002 and 0.007 for the timepoint I, II and III respectively. Several authors published guidelines for the effect size magnitude interpretation, in general an *ω*^2^≥ 0.15 is needed for a good (>0.8) statistical power [27,28], conversely, lower *ω*^2^ values indicate the need for a larger sample size. The estimations on the data from the present study showed that; whereas to evaluate the effect of the time factor a sample size of n = 5 is sufficient to obtain a power greater than 0.95 with an *ω*^2^ = 0.195 (Fig 5A), by contrast, considering the *ω*^2^ values obtained for the genotype effect, at least 20 samples for each group are needed (Fig 5B).

## Discussion

In our previous work [13] we showed the glycomic shift in the jejunal mucosa after the exposure to ETEC strains and we reported the influence of the porcine AO blood group genotype in these changes, suggesting that this host genetic background could affect the glycocalyx sugar motif and it may be relevant in the cross-talk between intestinal mucosa and bacterial community.

In the present study, the development of the faecal bacterial community from suckling to weaned piglets was investigated taking in to account the potential effect of the porcine AO blood type system. Furthermore, the absence of studies specifically focused on the blood groups/gut microbiota relations in pigs and the opposite conclusions reached by the two major studies concerning the association between ABO system and gut microbiota in humans [9,10] make the subject of study open to further insights.

The results clearly showed the dynamics of the modification in the faecal microbiota during weaning transition, illustrating that the experimental design has enough power to detect major microbiota differences. However, no evidences were reported for the influence of the blood types on bacterial community structure as well as in its specific taxa abundances. Nevertheless, we can hypotesize that changes in the mucosal glycomic pattern (proximal intestine) associated with the blood group genotypes may affect specific bacterial groups [8] but does not lead to changes that can affect the whole gut microbial community with such magnitude to be detectable by the current experimental design and the approach used for the analysis of the faecal microbiota [29]. Alternatively, we may speculate that in a given population with different genotypes related to different mucosal glycomic profiles and sharing the same environment, the best adapting way for the gut microbiota is to develop the ability to grow with no dependence for a specific intestinal sugar motif. In other words, the functional redundancy and the commensalism could represent an alternative to the competition to increase the fitness of the different microbial groups composing the gut microbiota [30], this would lead to a stable microbial community regardless of certain environmental variations such as different glycomic patterns of the intestinal mucosa.

We also tested the effect of the litter, on alpha and beta diversity of the bacterial communities, which can be a confounding factor (co-housing, maternal effect) in microbial community studies, but no significant differences associated with the litter effect were reported. The absence of this effect in previous studies was associated with the prevalence of stochastic factors in shaping the structure of early bacterial communities [5,31].

Focusing the attention on the adaptation to weaning transition, evident changes in the bacterial community were reported. In line with the literature [2,5,32], the alpha diversity values showed an increasing trend reaching values (timepoint III) comparable to that of the adult pigs microbiota (sows). The increasing values in alpha diversity are considered as a marker for a mature microbial community [33] and are associated to functional redundancy, which contributes to a greater stability of the microbial ecosystem in contrasting stressful events that may lead to dysbiotic conditions [34]. More generally, a greater variability within communities is positively correlated with the health status of the host [35]. It is interesting to note that in a recent study on miniature piglets [36] the alpha diversity decreased after weaning. As suggested by Chen [33], this may be due to different weaning ages in the different studies. Indeed, in the Hu’s study the piglets were weaned at 21 days of life (vs 28 days in our study). In general a greater weaning stress is associated with an earlier weaning age, thus this indicates that a more intense weaning stress may adversely affect the stability of the microbial community that is not yet ‘mature’ enough to face the new ecological conditions, promoting the proliferation of opportunistic pathobiont which can lead to typical disorders such as post-weaning diarrhoea [37].

The dynamic pattern of the inter-individual variation in bacterial community is well represented by the beta diversity results: the samples are clearly clustered for timepoint and increased distances among individuals in timepoint II (day 14) are observed which then converge to timepoint III (post-weaning) showing a greater uniformity among the microbial communities of the different individuals. The same pattern in beta diversity is shown in a larger longitudinal study [38], this variation may reflect the interindividual difference in intestinal maturation during the lactation period, which settles in the late post-weaning phase [39] allowing the establishment of a climax community [40].

Regarding the taxonomic shift, we found that the weaning transition is mainly characterized by a drastic reduction of Bacteroidaceae and Enterobacteriaceae paralleled by a dominance of Prevotellaceae in post-weaning, we also reported an increase in lactobacilli in post-weaning, but the differences were not significant. This taxonomic shift has been highlighted by several studies and it is generally correlated with the abrupt change from milk-based to cereal-based diet [5,32,41]. Indeed, Prevotellaceae is recognized as one of the families associated with the intake of fermentable fibers [42]. Studies on "milk-oriented microbiota" investigated the microbiome modifications in weaning transition focusing on the role of the sugar component of the diet. The rationale behind this hypothesis is that: the host, in proximal intestine, lacks of metabolic capacity to completely digest the different glycans, these glycans reach the distal intestine shaping the microbial community composition, hence, the gut microbiome of suckling piglets shows metabolisms oriented to the milk oligosaccharides consumption, whereas bacteria able to degrade plant-derived carbohydrates like Prevotellaceae became dominant after weaning [32,43]. This metabolic change was also reported by the metagenomic predictions in our study, showing the decrease in “Galactose metabolism” and the increase in “Starch and sucrose metabolism” after weaning transition. In addition, the metagenomic predictions of our study also showed a shift in bacterial lipid metabolism during the weaning transition. In particular, the predicted fatty acids degradative (*fad*) enzymes were enriched in the microbial communities of suckling piglets while the predicted fatty acid biosynthetic (*fab*) enzymes were enriched in the microbial communities of weaned piglets. It is known that the main source of energy in sow milk is fat, which mainly consists of long chain fatty acids [44], and it is also known that due to the lower pancreatic and intestinal lipase activities in the first part of the suckling period the nursing piglet does not have a complete ability to digest fat [45,46]; therefore, applying the same rationale used for glycans, we can hypothesize that un-digested fats can be used by intestinal bacteria capable of degrading fatty acids such as Enterobacteriaceae [47]. Conversely, the higher presence of enzymes involved in fatty acid biosynthesis [48], mainly due to Prevotellaceae, Ruminococcaceae and Lachnospiraceae in our study, can be linked to the higher fiber content in the post-weaning diet and to the ability to synthesize fatty acids by fermentation of complex carbohydrates of these bacteria. Although it is not easy to define the role of fats in microbial community modulation, there are some evidence that indicate the levels of fat in sow milk as one of the factors that may influence the composition of the faecal microbiota of the piglets [38]. In addition, a recent study on germ-free mice inoculated with faecal microbiota from breast-fed infants showed that the administration of long chain fatty acid-rich emulsions resulted in an increase in Enterobacteriaceae, while the administration of medium chain fatty acid-rich emulsions resulted in an increase in Bacteroidaceae in faecal bacterial community of mice [49].

Furthermore, it has been reported that in *E*. *coli* the *fad* enzymes are induced by the long chain fatty acids but not by the short and medium chain fatty acids [47] and that the genes fadJ and fadI, involved in anaerobic utilization of fatty acids, may play a key role in *E*. *coli* pathogenesis in environments with low oxygen tensions [50], therefore, the use of milk-derived long chain fatty acids as an energy source could represent an opportunistic strategy used by *E*. *coli* strains during the lactation period.

In conclusion, the present study showed the changes in faecal microbiota during the weaning transition in pigs, suggesting a role of the fatty component of sow milk in the selection of Enterobacteriaceae in the gut bacterial community of the suckling piglets. Although supported by the literature, this hypothesis is based on metagenomic predictions and this represents a limitation of the present study, hence, specific studies aimed at testing it are needed.

On the other hand, no evidences in favour of the hypothesis that the genotypes for the porcine AO blood groups can affect the faecal bacterial community composition of the piglets were reported. However, as shown by the results concerning the statistical power, the sample size of the present study could represent a limiting factor for the identification of genotype effects on faecal microbial communities. Thus, larger studies and aimed at analyzing the intestinal rather than the faecal microbiota could be useful to better examine this hypothesis that is still the subject of discussion in various fields.

## Supporting information

S1 Table Primer used for blood groups AO genotypes screening. (DOCX)

S2 Table Per sample metadata, sequencing yield, OTUs abundances and Shannon index values. (DOCX)

S1 Models Models fitted in nlme package to test the effect of genotype and litter factors on alpha diversity (Shannon index) in piglets. (DOCX)

S2 Models Models fitted with Adonis procedure in vegan package to test the effect of genotype and litter factors on beta diversity (Bray-Curtis distance) in piglets. (DOCX)

S1 Fig Per sample rarefaction curves. p = piglets, S = Sows, I = day7 post farrowing, II = day14 post farrowing, III = day14 post weaning.
(TIF)

S2 Fig Per sample taxonomic classification top 5 Phyla (relative abundances). p = piglets, S = Sows, I = day7 post farrowing, II = day14 post farrowing, III = day14 post weaning.
(TIF)

S3 Fig Per sample taxonomic classification top 10 Families (relative abundances). p = piglets, S = Sows, I = day7 post farrowing, II = day14 post farrowing, III = day14 post weaning.
(TIF)

S4 Fig Per sample taxonomic classification top 10 Genera (relative abundances). p = piglets, S = Sows, I = day7 post farrowing, II = day14 post farrowing, III = day14 post weaning.
(TIF)

S5 Fig Non-metric multidimensional scaling (NMDS) on Bray-Curtis distances at Family level. pI = piglets timepoint I (day 7 post farrowing), pII = piglets timepoint II (day 14 post farrowing), pIII = piglets timepoint III (day 14 post weaning), SI = sows timepoint I (day 7 post farrowing) SII = sows timepoint II (day 14 post farrowing).
(TIF)

S6 Fig Extended error bar plot showing the families that have significantly different abundances between piglets at the timepoint I and piglets at the timepoint II. pI = piglets timepoint I (day 7 post farrowing), pII = piglets timepoint II (day 14 post farrowing). The differences were tested in metaGenomeseq package as reported in Methods section.
(TIF)

S7 Fig Extended error bar plot showing the families that have significantly different abundances between suckling piglets (pre_W) and weaned piglets (post_W). pre_W = piglets timepoint I (day 7 post farrowing) + piglets timepoint II (day 14 post farrowing), post_W = piglets timepoint III (day 14 post weaning). The differences were tested in metaGenomeseq package as reported in Methods section.
(TIF)

S8 Fig Extended error bar plot showing the families that have significantly different abundances between Sows (M) and weaned piglets (post_W). M = mature microbiota (Sows day 7 post farrowing + sows day 14 post farrowing), post_W = piglets timepoint III (day 14 post weaning). The differences were tested in metaGenomeseq package as reported in Methods section.
(TIF)

S9 Fig Extended error bar plot showing the Level3 KEEG pathways that have significantly different abundances between piglets at the timepoint I and piglets at the timepoint II. pI = piglets timepoint I (day 7 post farrowing), pII = piglets timepoint II (day 14 post farrowing). The differences were tested in STAMP as reported in M&M section. Most of these pathways have not biological meaning for prokaryotes.
(TIF)

S10 Fig Boxplot showing the differences in “Fatty acid metabolism” (Level3 KEEG pathway) between suckling piglets (pre_W) and weaned piglets (post_W). pre_W = piglets timepoint I (day 7 post farrowing) + piglets timepoint II (day 14 post farrowing), post_W = piglets timepoint III (day 14 post weaning).The differences were tested in STAMP as reported in Methods section.
(TIF)

S11 Fig Boxplot showing the differences in “Galactose metabolism” (Level3 KEEG pathway) between suckling piglets (pre_W) and weaned piglets (post_W). pre_W = piglets timepoint I (day 7 post farrowing) + piglets timepoint II (day 14 post farrowing), post_W = piglets timepoint III (day 14 post weaning). The differences were tested in STAMP as reported in Methods section.
(TIF)

S12 Fig Boxplot showing the differences in “Starch and sucrose metabolism” (Level3 KEEG pathway) between suckling piglets (pre_W) and weaned piglets (post_W). pre_W = piglets timepoint I (day 7 post farrowing) + piglets timepoint II (day 14 post farrowing), post_W = piglets timepoint III (day 14 post weaning). The differences were tested in STAMP as reported in Methods section.
(TIF)

S13 Fig IPath2 metabolic map. in green are highlighted the pathways significantly enriched in bacterial community of suckling piglets, in green are highlighted the pathways significantly enriched in bacterial community of weaned piglets. The differences were tested in DeSeq2 package as reported in Methods section.
(TIF)

S14 Fig Per sample taxonomic classification of Genera within Enterobacteriaceae family (relative abundances). Taxonomic refinement by DADA2 pipeline with SILVA database. p = piglets, S = Sows, I = day7 post farrowing, II = day14 post farrowing, III = day14 post weaning.
(TIF)
