# Supplementary material for: Faecal microbiota shift during weaning transition in piglets and evaluation of AO blood types as shaping factor for the bacterial community profile
Source: PLoS One. 2019 May 16;14(5):e0217001. doi: 10.1371/journal.pone.0217001 (PMC6522051; doi:10.1371/journal.pone.0217001)
Supplement: S1 Table — (DOCX) [file pone.0217001.s001.docx]

**S1 Table. Primer used for blood groups A0 genotypes screening.**

| Primer sequence (5'->3') | | Amplicon length | Accession number | Reference |
| --- | --- | --- | --- | --- |
| Forward | CGCCAGTCCTTCACCTACGAAC | 1076 -> allele 0 | GU256574 | Nguyen et al. (2011) |
| Reverse | CGGTTCCGAATCTCTGCGTG |  |  |  |
| Forward | AATGTCCTTATGCTGGCCTGG | 1076 and 340 -> allele A | GU256573 |  |
| Reverse | AACAACACACTCCTGAACAACAGA |  |  |  |
