# Supplementary material for: Faecal microbiota shift during weaning transition in piglets and evaluation of AO blood types as shaping factor for the bacterial community profile
Source: PLoS One. 2019 May 16;14(5):e0217001. doi: 10.1371/journal.pone.0217001 (PMC6522051; doi:10.1371/journal.pone.0217001)
Supplement: S2 Table — (DOCX) [file pone.0217001.s002.docx]

**S2 Table.** **Per sample metadata, sequencing yield, OTUs abundances and Shannon index values.**

| **Subject** | **Timepoint**^a^ | **Category**^b^ | **Genotype** | **Litter** | **Reads**  **count** | **Observed OTUs** | **Shannon** |
| --- | --- | --- | --- | --- | --- | --- | --- |
| 216 | I | p | AO | S2 | 47,878 | 394 | 3.04 |
| 211 | I | p | AO | S2 | 63,472 | 466 | 3.80 |
| 212 | I | p | AO | S2 | 59,272 | 456 | 3.90 |
| 227 | I | p | AO | S3 | 136,943 | 486 | 4.32 |
| 224 | I | p | AO | S3 | 69,831 | 311 | 3.42 |
| 237 | I | p | OO | S1 | 100,801 | 369 | 3.80 |
| 236 | I | p | OO | S1 | 115,145 | 384 | 3.19 |
| 254 | I | p | OO | S4 | 126,739 | 485 | 3.46 |
| 257 | I | p | OO | S4 | 42,252 | 325 | 3.64 |
| 256 | I | p | OO | S4 | 51,381 | 368 | 3.74 |
| 211 | II | p | AO | S2 | 80,263 | 534 | 3.77 |
| 212 | II | p | AO | S2 | 95,286 | 464 | 3.78 |
| 216 | II | p | AO | S2 | 88,498 | 539 | 3.68 |
| 231 | II | p | AO | S3 | 94,577 | 533 | 3.73 |
| 224 | II | p | AO | S3 | 44,729 | 594 | 3.80 |
| 227 | II | p | AO | S3 | 68,069 | 665 | 4.64 |
| 238 | II | p | OO | S1 | 94,572 | 467 | 4.04 |
| 237 | II | p | OO | S1 | 52,949 | 597 | 4.58 |
| 236 | II | p | OO | S1 | 73,944 | 487 | 4.17 |
| 256 | II | p | OO | S4 | 107,435 | 426 | 4.08 |
| 254 | II | p | OO | S4 | 110,306 | 343 | 3.00 |
| 257 | II | p | OO | S4 | 40,715 | 325 | 3.46 |
| 216 | III | p | AO | S2 | 60,494 | 734 | 4.77 |
| 211 | III | p | AO | S2 | 87,581 | 732 | 4.61 |
| 212 | III | p | AO | S2 | 52,175 | 602 | 4.57 |
| 231 | III | p | AO | S3 | 58,497 | 786 | 5.01 |
| 224 | III | p | AO | S3 | 61,965 | 832 | 4.96 |
| 227 | III | p | AO | S3 | 65,919 | 766 | 4.81 |
| 237 | III | p | OO | S1 | 69,650 | 837 | 5.13 |
| 238 | III | p | OO | S1 | 95,203 | 923 | 5.07 |
| 236 | III | p | OO | S1 | 66,477 | 750 | 4.88 |
| 256 | III | p | OO | S4 | 66,259 | 879 | 5.18 |
| 254 | III | p | OO | S4 | 94,765 | 863 | 5.03 |
| 257 | III | p | OO | S4 | 120,230 | 846 | 5.30 |
| 135128 | I | S | AO | S2 | 148,653 | 750 | 4.42 |
| 135130 | I | S | AO | S3 | 135,259 | 777 | 4.88 |
| 135120 | I | S | OO | S1 | 117,618 | 818 | 4.72 |
| 135136 | I | S | OO | S4 | 77,230 | 753 | 4.86 |
| 135128 | II | S | AO | S2 | 69,160 | 745 | 5.18 |
| 135130 | II | S | AO | S3 | 62,834 | 683 | 5.02 |
| 135120 | II | S | OO | S1 | 100,383 | 759 | 4.55 |
| 135136 | II | S | OO | S4 | 70,559 | 725 | 4.42 |

^a^I= 7 days post-farrowing, II= 14 days post-farrowing, III= 14 days post-weaning;

^b^p= piglet; S= sow
