# Supplementary material for: Faecal microbiota shift during weaning transition in piglets and evaluation of AO blood types as shaping factor for the bacterial community profile
Source: PLoS One. 2019 May 16;14(5):e0217001. doi: 10.1371/journal.pone.0217001 (PMC6522051; doi:10.1371/journal.pone.0217001)
Supplement: S1 Models — (DOCX) [file pone.0217001.s003.docx]

**S1 Models.** Models fitted in nlme package to test the effect of genotype and litter factors on alpha diversity (Shannon index) in piglets.

Shannon Piglets ~ Genotype, random = ~ 1|Timepoint/Subject

|  | numDF | denDF | F-value | p-value |
| --- | --- | --- | --- | --- |
| (Intercept) | 1 | 30 | 107.56 | <.0001 |
| Genotype | 1 | 30 | 0.30 | 0.5861 |

Shannon Piglets ~ Litter, random = ~ 1|Timepoint/Subject

|  | numDF | denDF | F-value | p-value |
| --- | --- | --- | --- | --- |
| (Intercept) | 1 | 28 | 110.38 | <.0001 |
| Litter | 3 | 28 | 1.43 | 0.2537 |

Shannon Piglets ~ Litter, random = ~ 1|Timepoint/Genotype/Subject

|  | numDF | denDF | F-value | p-value |
| --- | --- | --- | --- | --- |
| (Intercept) | 1 | 25 | 110.38 | <.0001 |
| Litter | 3 | 25 | 1.44 | 0.2557 |
