# Supplementary material for: Faecal microbiota shift during weaning transition in piglets and evaluation of AO blood types as shaping factor for the bacterial community profile
Source: PLoS One. 2019 May 16;14(5):e0217001. doi: 10.1371/journal.pone.0217001 (PMC6522051; doi:10.1371/journal.pone.0217001)
Supplement: S2 Models — (DOCX) [file pone.0217001.s004.docx]

**S2 Models.** Models fitted with Adonis procedure in vegan package to test the effect of genotype and litter factors on beta diversity (Bray-Curtis distance) in piglets.

Bray-Curtis Piglets ~ Timepoint

|  | Df | SumsOfSqs | MeanSqs | F.Model | R2 | Pr(>F) |
| --- | --- | --- | --- | --- | --- | --- |
| Maturity | 2 | 4.2092 | 2.10460 | 8.7301 | 0.3603 | 0.001 |
| Residuals | 31 | 7.4733 | 0.24107 |  | 0.6397 |  |
| Total | 32 | 11.6825 |  |  | 1.0000 |  |

Bray-Curtis Piglets ~ Litter, strata = Timepoint

|  | Df | SumsOfSqs | MeanSqs | F.Model | R2 | Pr(>F) |
| --- | --- | --- | --- | --- | --- | --- |
| Litter | 3 | 0.864 | 0.28801 | 0.79866 | 0.07369 | 0.090 |
| Residuals | 30 | 10.819 | 0.36062 |  | 0.92604 |  |
| Total | 33 | 11.682 |  |  | 1.00000 |  |

Bray-Curtis Piglets ~ Genotype, strata = Timepoint

|  | Df | SumsOfSqs | MeanSqs | F.Model | R2 | Pr(>F) |
| --- | --- | --- | --- | --- | --- | --- |
| Genotype | 1 | 0.2597 | 0.25967 | 0.72743 | 0.02223 | 0.333 |
| Residuals | 32 | 11.4228 | 0.35696 |  | 0.97777 |  |
| Total | 33 | 11.6825 |  |  | 1.0000 |  |

Bray-Curtis Piglets ~ Litter %in% Genotype, strata = Timepoint

|  | Df | SumsOfSqs | MeanSqs | F.Model | R2 | Pr(>F) |
| --- | --- | --- | --- | --- | --- | --- |
| Litter: Genotype | 3 | 0.864 | 0.28801 | 0.79866 | 0.07369 | 0.117 |
| Residuals | 30 | 10.819 | 0.36062 |  | 0.92604 |  |
| Total | 33 | 11.682 |  |  | 1.00000 |  |
