# Supplementary figures and images for: Faecal microbiota shift during weaning transition in piglets and evaluation of AO blood types as shaping factor for the bacterial community profile
Source: PLoS One. 2019 May 16;14(5):e0217001. doi: 10.1371/journal.pone.0217001 (PMC6522051; doi:10.1371/journal.pone.0217001)

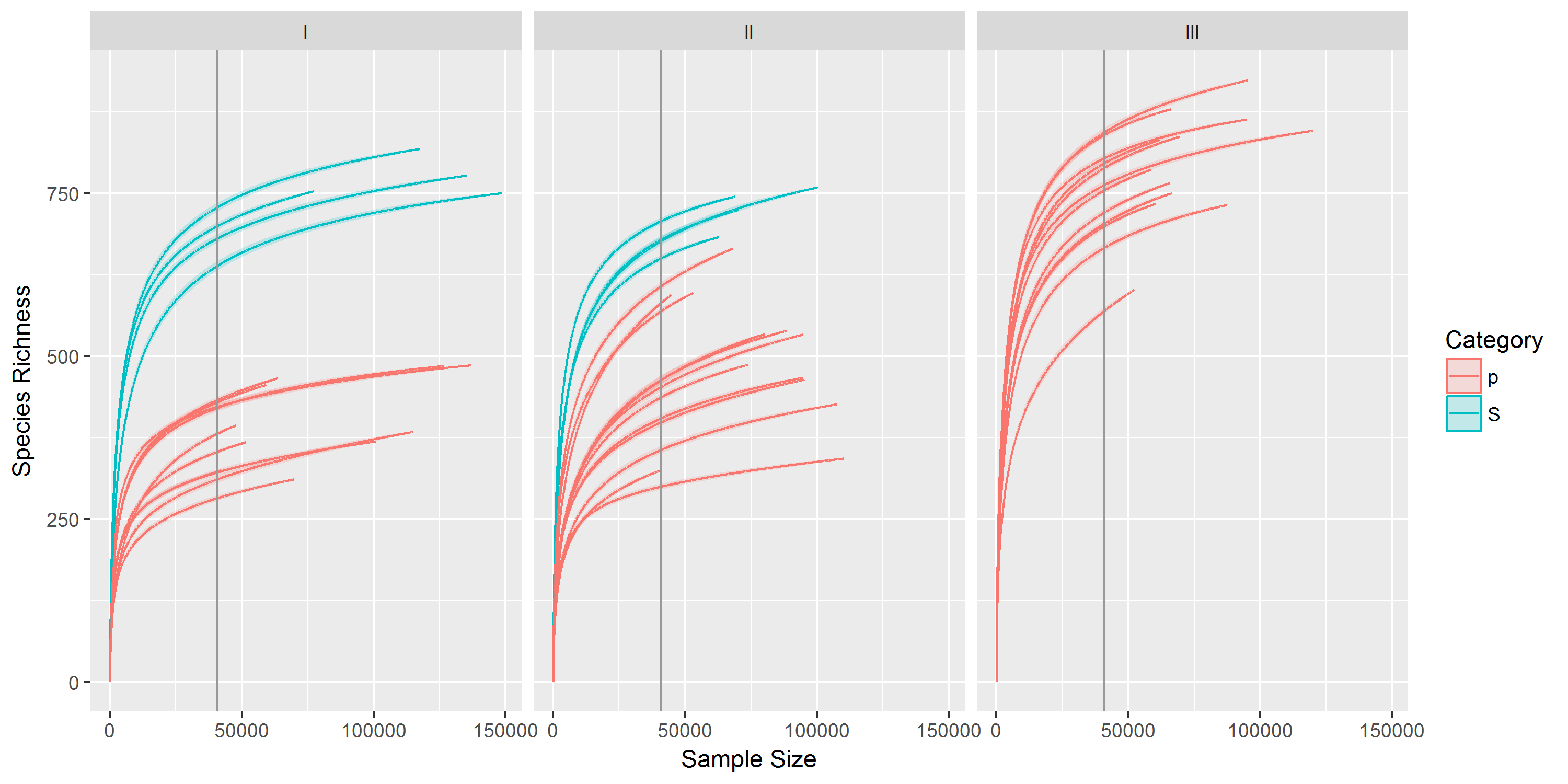

Supplement: S1 Fig — p = piglets, S = Sows, I = day7 post farrowing, II = day14 post farrowing, III = day14 post weaning. (TIF) [file pone.0217001.s005.tif]

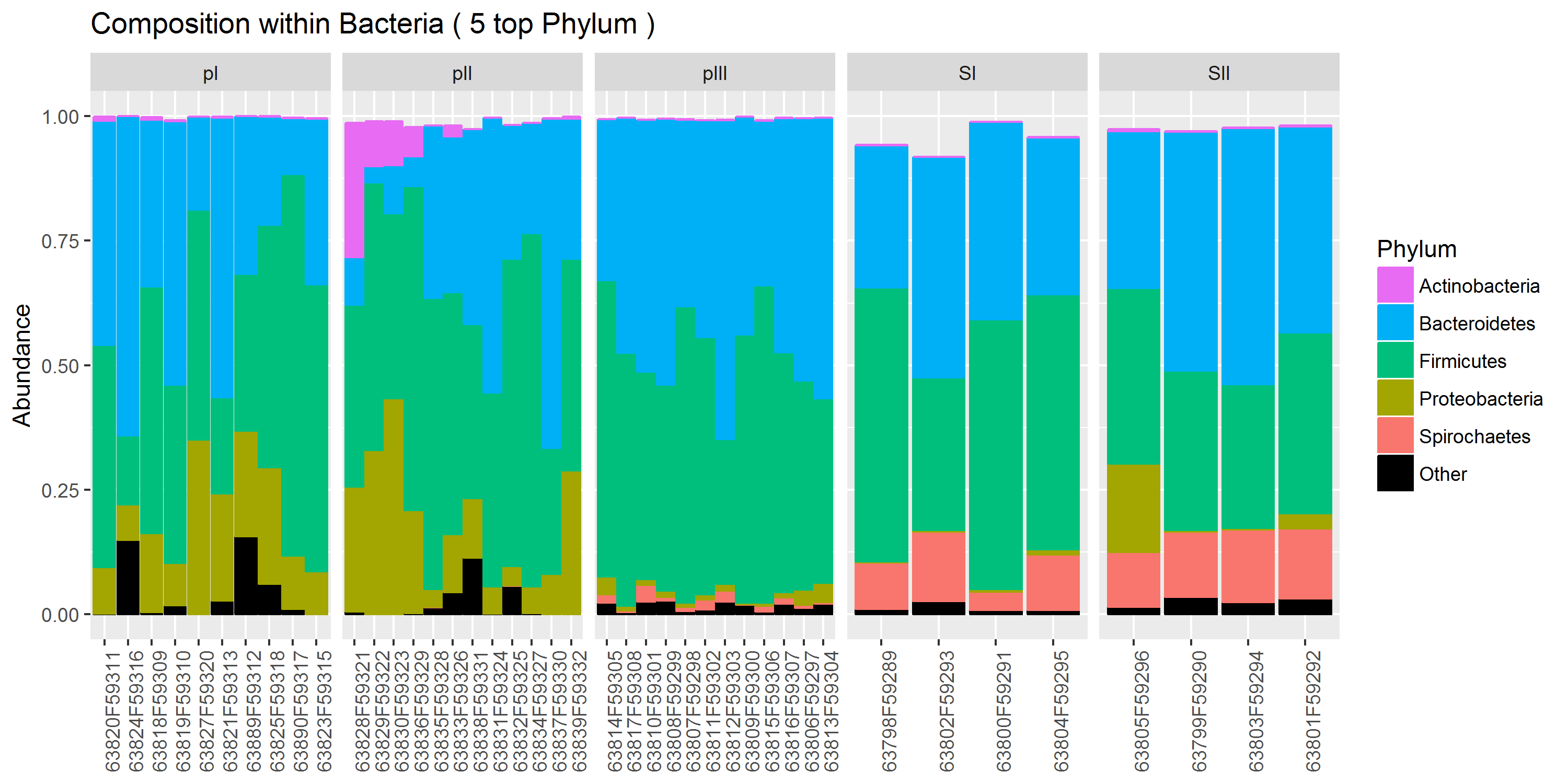

Supplement: S2 Fig — p = piglets, S = Sows, I = day7 post farrowing, II = day14 post farrowing, III = day14 post weaning. (TIF) [file pone.0217001.s006.tif]

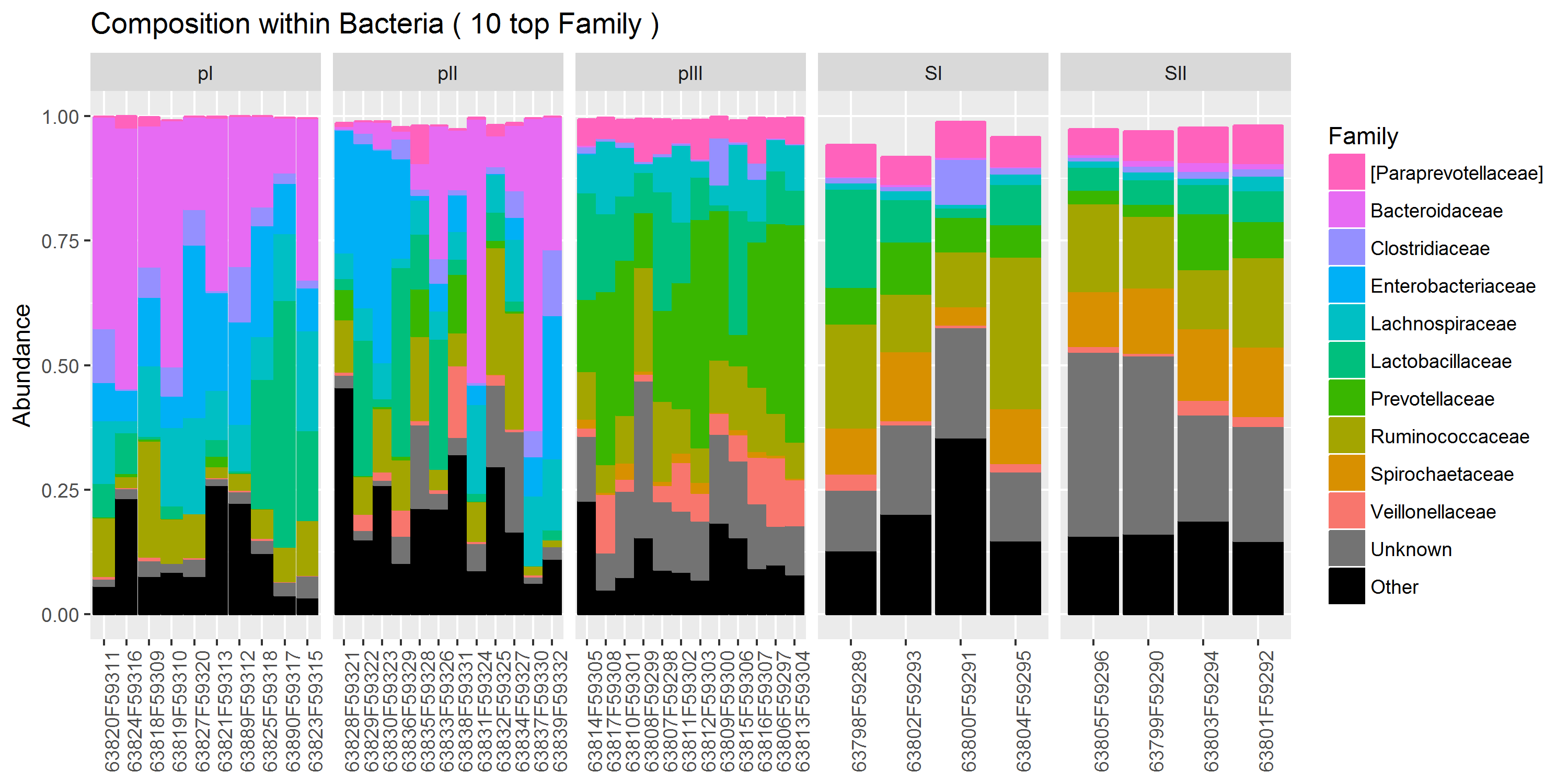

Supplement: S3 Fig — p = piglets, S = Sows, I = day7 post farrowing, II = day14 post farrowing, III = day14 post weaning. (TIF) [file pone.0217001.s007.tif]

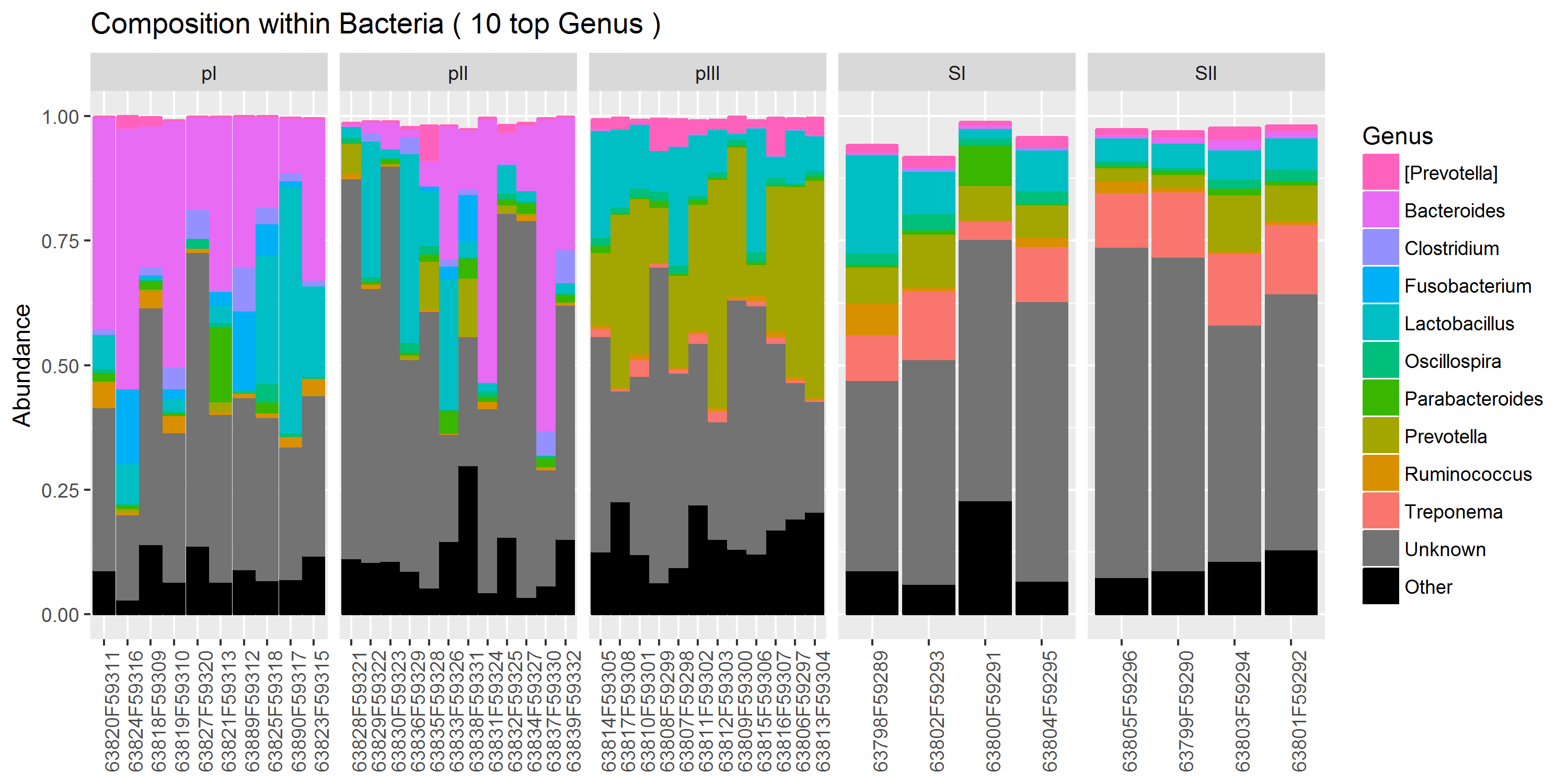

Supplement: S4 Fig — p = piglets, S = Sows, I = day7 post farrowing, II = day14 post farrowing, III = day14 post weaning. (TIF) [file pone.0217001.s008.tif]

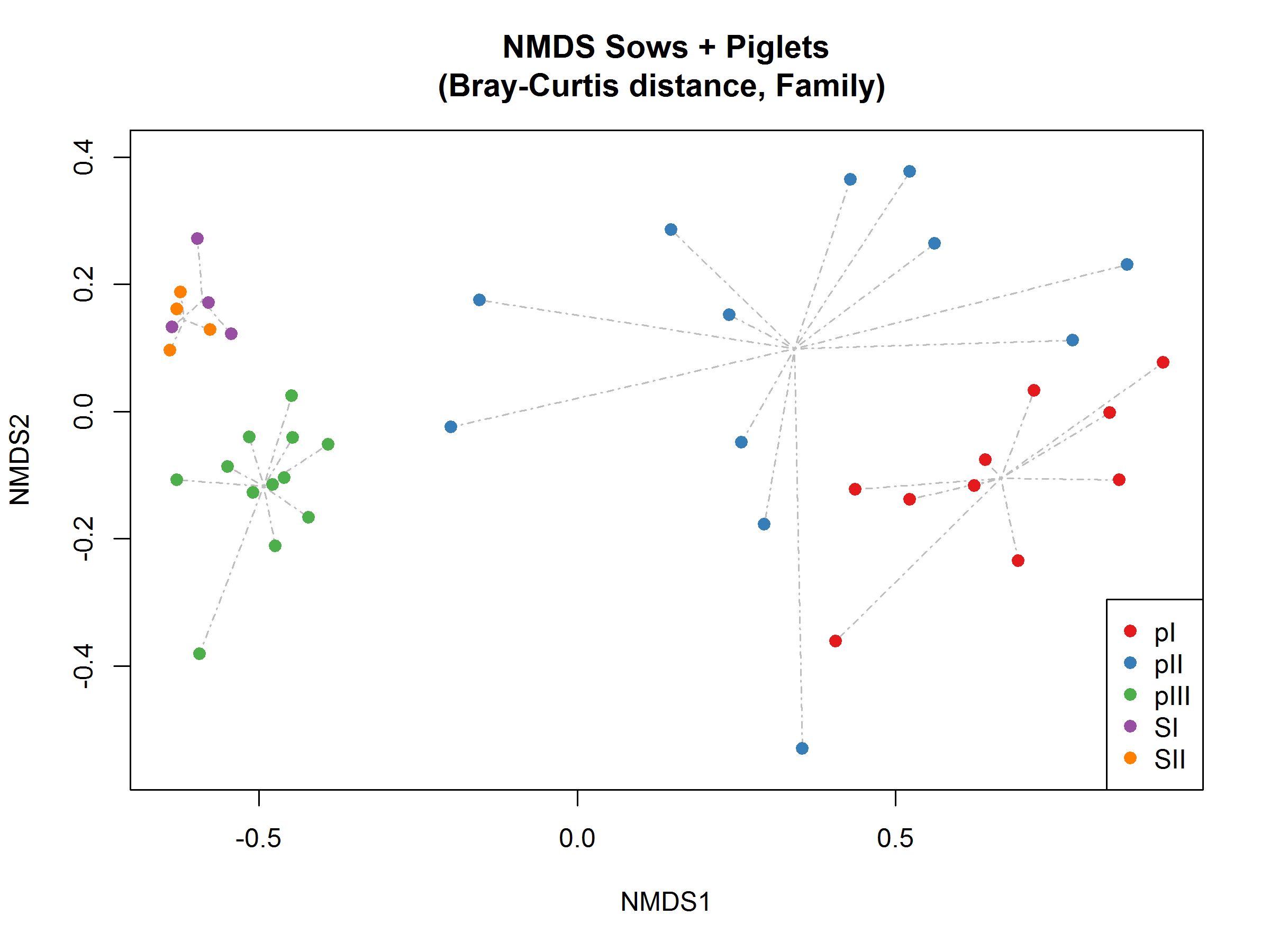

Supplement: S5 Fig — pI = piglets timepoint I (day 7 post farrowing), pII = piglets timepoint II (day 14 post farrowing), pIII = piglets timepoint III (day 14 post weaning), SI = sows timepoint I (day 7 post farrowing) SII = sows timepoint II (day 14 post farrowing). (TIF) [file pone.0217001.s009.tif]

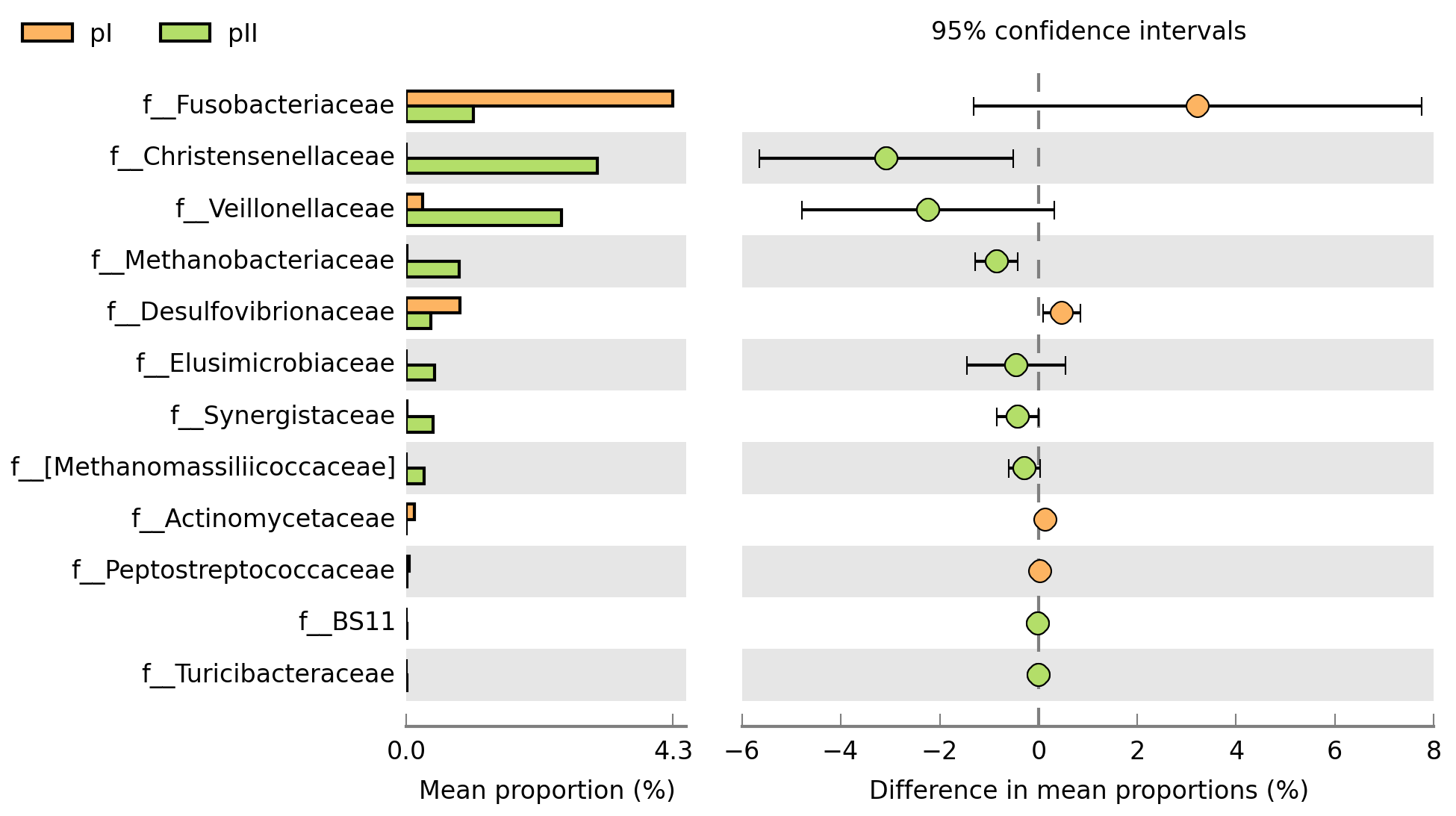

Supplement: S6 Fig — pI = piglets timepoint I (day 7 post farrowing), pII = piglets timepoint II (day 14 post farrowing). The differences were tested in metaGenomeseq package as reported in Methods section. (TIF) [file pone.0217001.s010.tif]

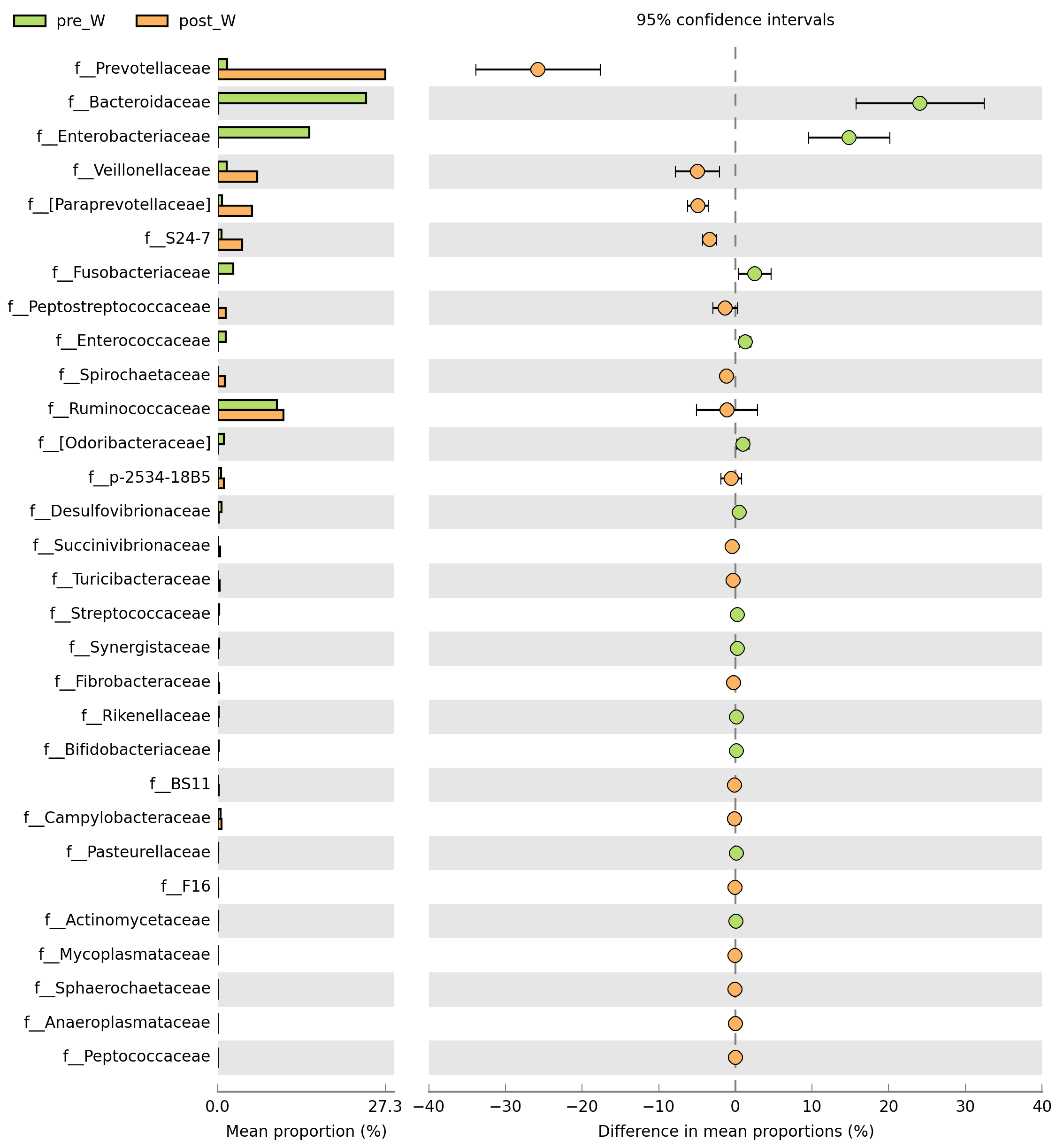

Supplement: S7 Fig — pre_W = piglets timepoint I (day 7 post farrowing) + piglets timepoint II (day 14 post farrowing), post_W = piglets timepoint III (day 14 post weaning). The differences were tested in metaGenomeseq package as reported in Methods section. (TIF) [file pone.0217001.s011.tif]

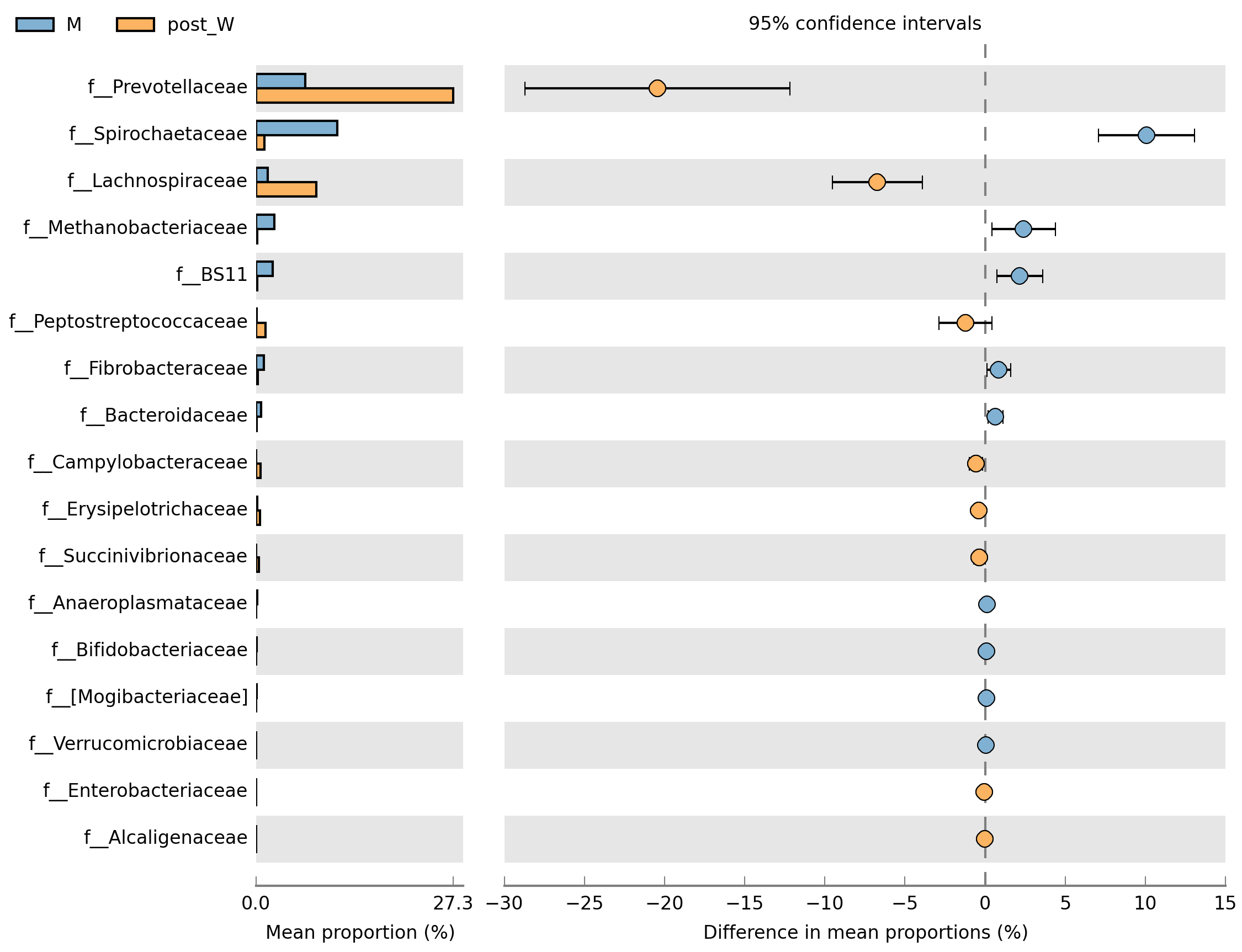

Supplement: S8 Fig — M = mature microbiota (Sows day 7 post farrowing + sows day 14 post farrowing), post_W = piglets timepoint III (day 14 post weaning). The differences were tested in metaGenomeseq package as reported in Methods section. (TIF) [file pone.0217001.s012.tif]

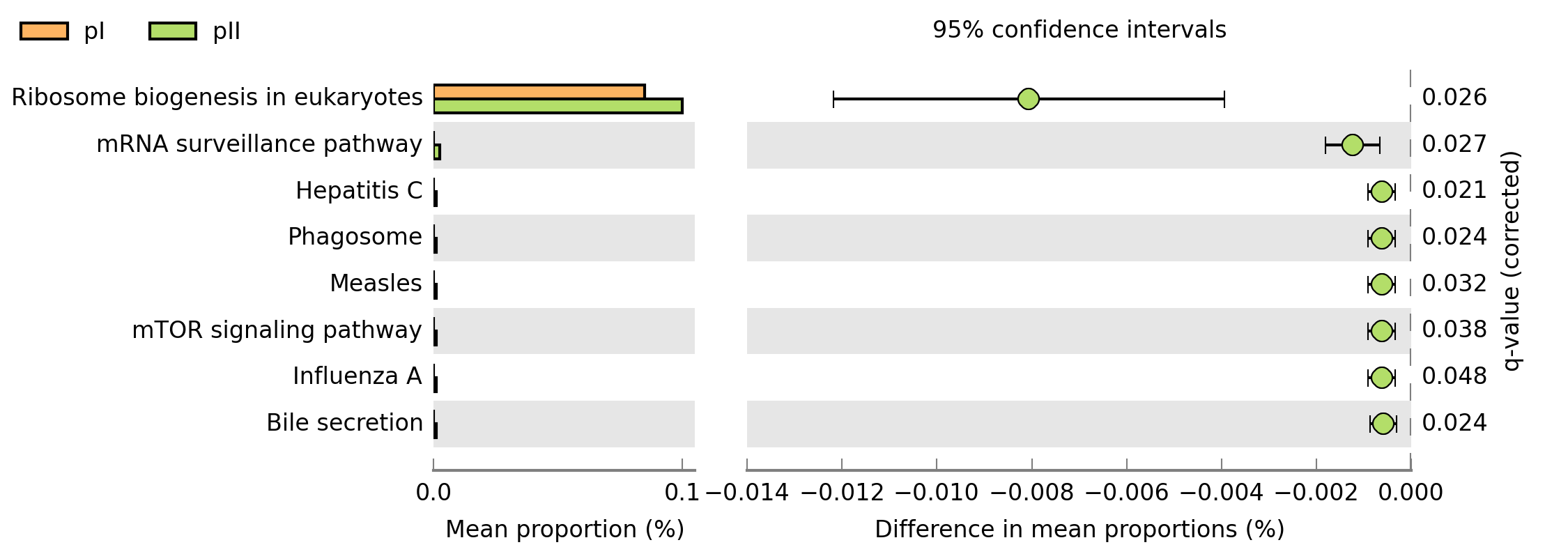

Supplement: S9 Fig — pI = piglets timepoint I (day 7 post farrowing), pII = piglets timepoint II (day 14 post farrowing). The differences were tested in STAMP as reported in M&M section. Most of these pathways have not biological meaning for prokaryotes. (TIF) [file pone.0217001.s013.tif]

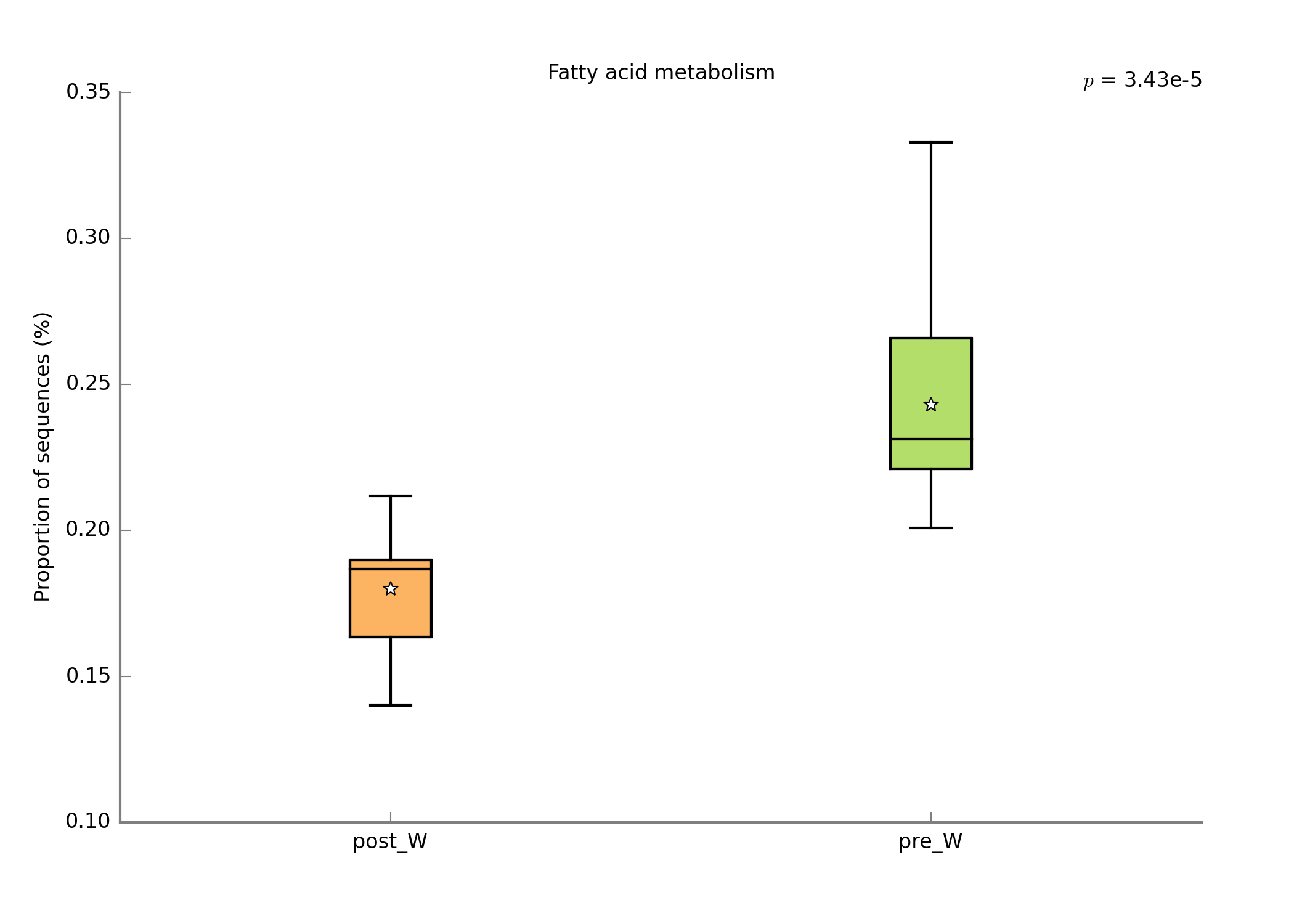

Supplement: S10 Fig — pre_W = piglets timepoint I (day 7 post farrowing) + piglets timepoint II (day 14 post farrowing), post_W = piglets timepoint III (day 14 post weaning).The differences were tested in STAMP as reported in Methods section. (TIF) [file pone.0217001.s014.tif]

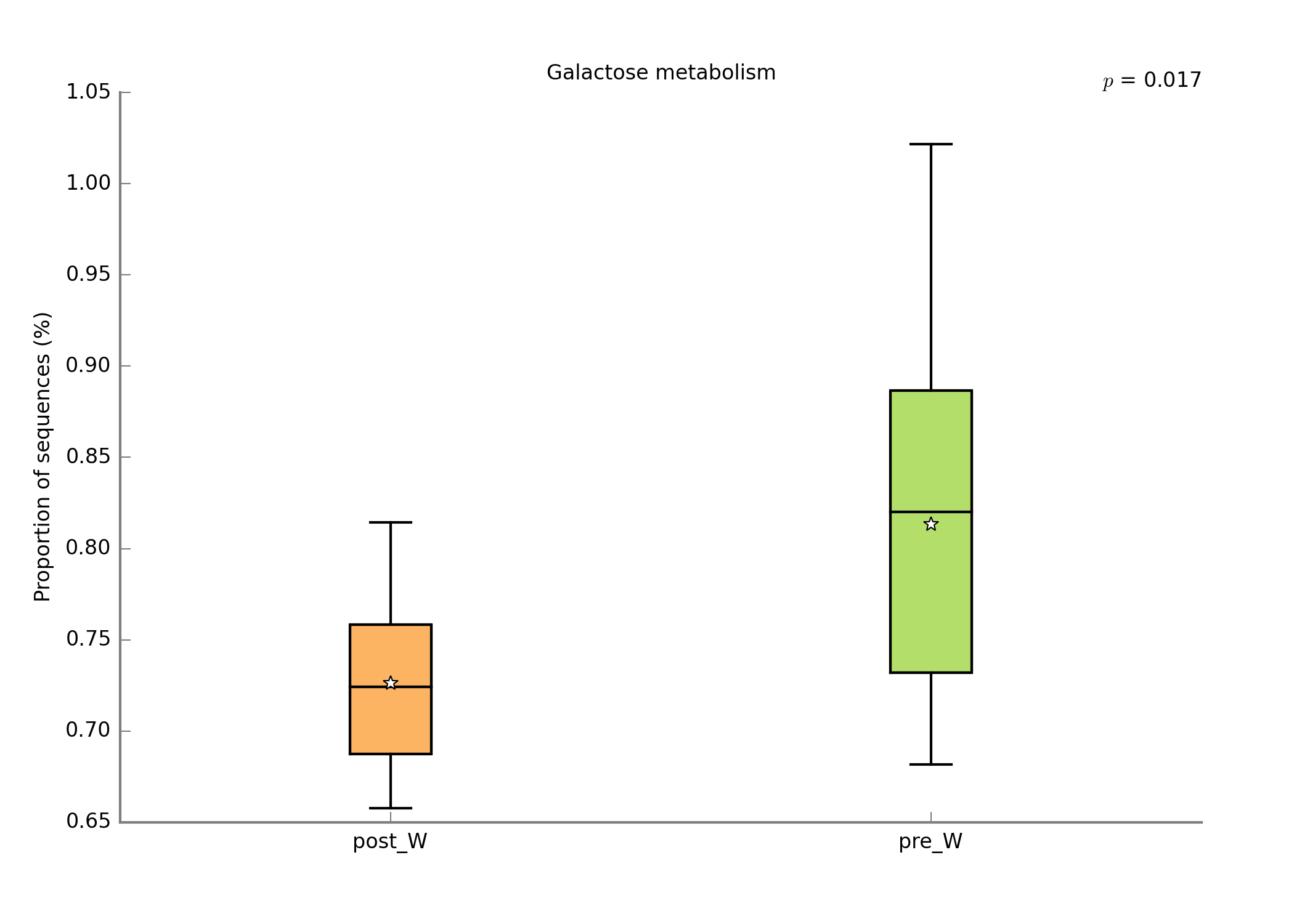

Supplement: S11 Fig — pre_W = piglets timepoint I (day 7 post farrowing) + piglets timepoint II (day 14 post farrowing), post_W = piglets timepoint III (day 14 post weaning). The differences were tested in STAMP as reported in Methods section. (TIF) [file pone.0217001.s015.tif]

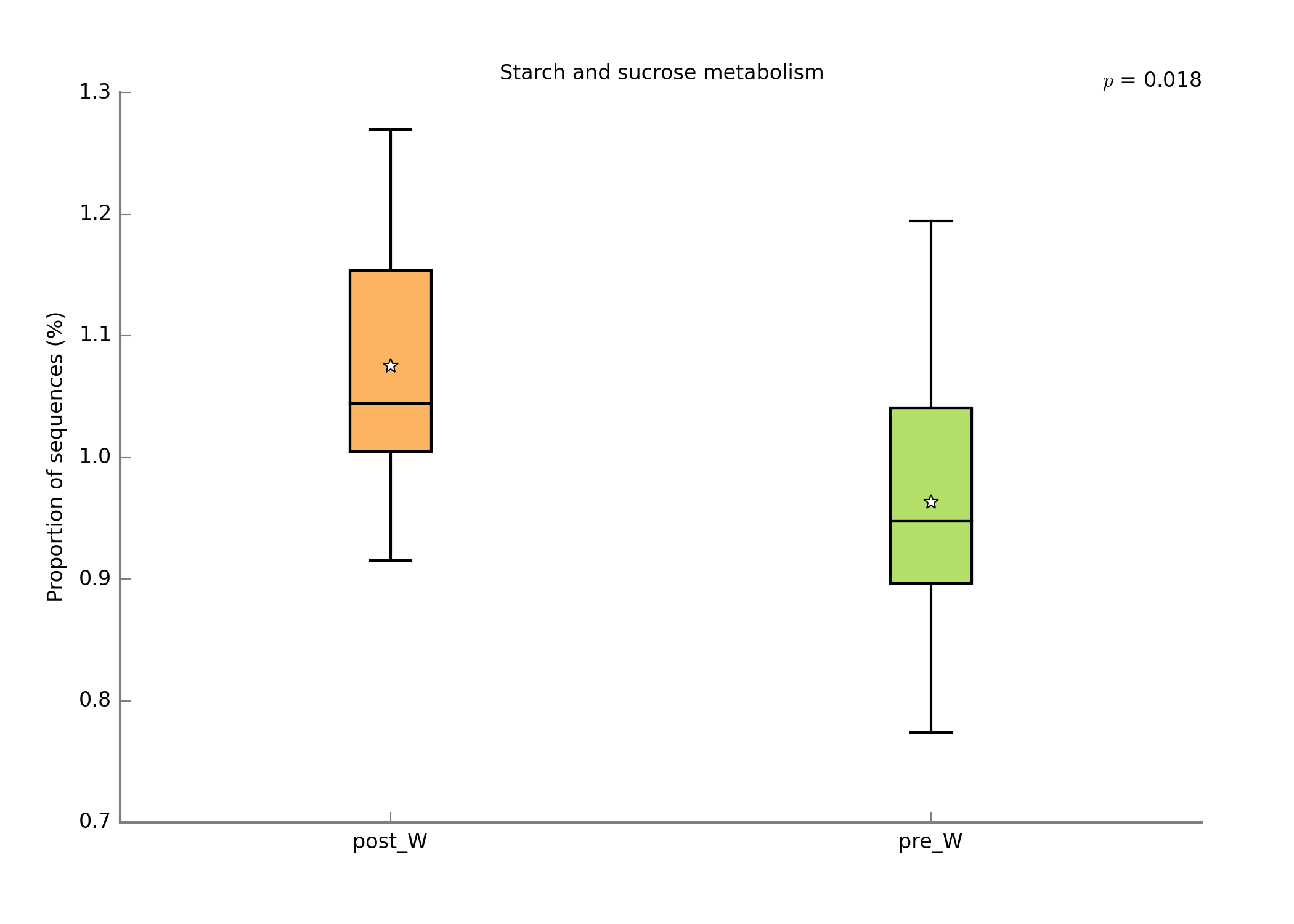

Supplement: S12 Fig — pre_W = piglets timepoint I (day 7 post farrowing) + piglets timepoint II (day 14 post farrowing), post_W = piglets timepoint III (day 14 post weaning). The differences were tested in STAMP as reported in Methods section. (TIF) [file pone.0217001.s016.tif]

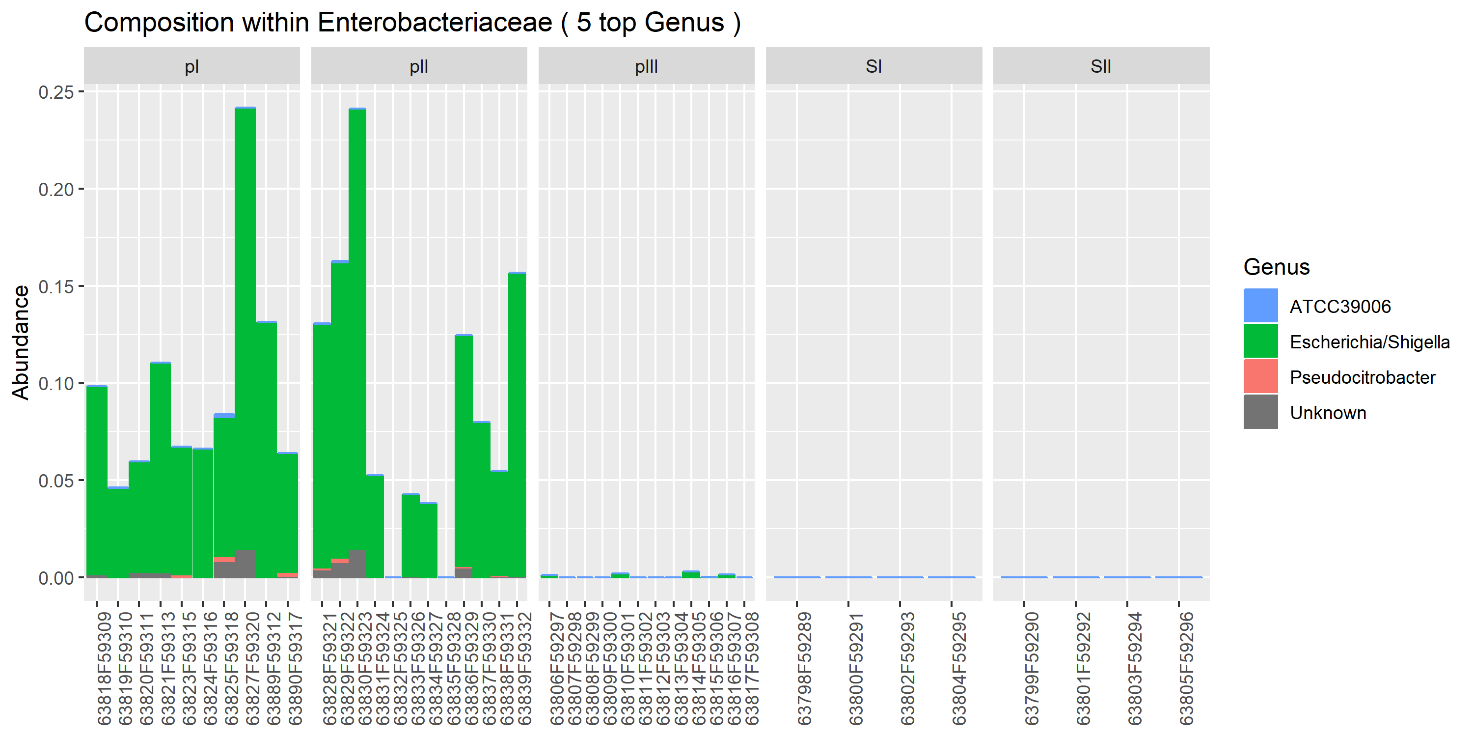

Supplement: S14 Fig — p = piglets, S = Sows, I = day7 post farrowing, II = day14 post farrowing, III = day14 post weaning. (TIF) [file pone.0217001.s018.tif]
